# Genomic and antigenic diversity of colonizing *Klebsiella pneumoniae* isolates mirrors that of invasive isolates in Blantyre, Malawi

**DOI:** 10.1099/mgen.0.000778

**Published:** 2022-03-18

**Authors:** Joseph M. Lewis, Madalitso Mphasa, Rachel Banda, Mathew A. Beale, Jane Mallewa, Eva Heinz, Nicholas R. Thomson, Nicholas A. Feasey

**Affiliations:** ^1^​ Malawi-Liverpool Wellcome Research Programme, Kamuzu University of Health Sciences, Blantyre, Malawi; ^2^​ Liverpool School of Tropical Medicine, Liverpool, UK; ^3^​ University of Liverpool, Liverpool, UK; ^4^​ Wellcome Sanger Institute, Hinxton, UK; ^5^​ Kamuzu University of Health Sciences, Blantyre, Malawi; ^6^​ London School of Hygiene and Tropical Medicine, London, UK

**Keywords:** whole-genome sequencing, Africa south of the Sahara, drug resistance, microbial, extended-spectrum beta-lactamase

## Abstract

Members of the *Klebsiella pneumoniae* species complex, particularly *K. pneumoniae* subsp. *pneumoniae* are antimicrobial resistance (AMR) associated pathogens of global importance, and polyvalent vaccines targeting *Klebsiella* O-antigens are in development. Whole-genome sequencing has provided insight into O-antigen distribution in the *K. pneumoniae* species complex*,* as well as population structure and virulence determinants, but genomes from sub-Saharan Africa are underrepresented in global sequencing efforts. We therefore carried out a genomic analysis of extended-spectrum beta-lactamase (ESBL)-producing *K. pneumoniae* species complex isolates colonizing adults in Blantyre, Malawi. We placed these isolates in a global genomic context, and compared colonizing to invasive isolates from the main public hospital in Blantyre. In total, 203 isolates from stool and rectal swabs from adults were whole-genome sequenced and compared to a publicly available multicounty collection and previously sequenced Malawian and Kenyan isolates from blood or sterile sites. We inferred phylogenetic relationships and analysed the diversity of genetic loci linked to AMR, virulence, capsule and LPS O-antigen (O-types). We find that the diversity of Malawian *K. pneumoniae* subsp. *pneumoniae* isolates represents the species’ population structure, but shows distinct local signatures concerning clonal expansions. Siderophore and hypermucoidy genes were more frequent in invasive versus colonizing isolates (present in 13 % vs 1 %) but still generally lacking in most invasive isolates. O-antigen population structure and distribution was similar in invasive and colonizing isolates, with O4 more common (14%) than in previously published studies (2–5 %). We conclude that host factors, pathogen opportunity or alternate virulence loci not linked to invasive disease elsewhere are likely to be the major determinants of invasive disease in Malawi. Distinct ST and O-type distributions in Malawi highlight the need to sample locations where the burden of invasive *Klebsiella* disease is greatest to robustly define secular trends in *Klebsiella* diversity to assist in the development of a useful vaccine. Colonizing and invasive isolates in Blantyre are similar, hence O-typing of colonizing *Klebsiella* isolates may be a rapid and cost-effective approach to describe global diversity and guide vaccine development.

## Impact Statement

The *Klebsiella pneumoniae* species complex is a major cause of drug-resistant infections worldwide, and whole-genome sequencing has started to provide important insights into its global distribution. However, most sequenced *Klebsiella* genomes have been collected from high-income settings, and isolates from low- and middle-income settings, especially the nations of sub-Saharan Africa, are underrepresented. Here we begin to address that by sequencing 203 ESBL *K. pneumoniae* complex isolates colonising adults in Blantyre, Malawi, and comparing them to previously sequenced invasive Malawian isolates as well as global collections. We find locally circulating successful lineages of *K. pneumoniae* subsp. *pneumoniae* in Malawi, which may be different to successful lineages elsewhere; understanding the reasons for this success could help to understand transmission routes. *Klebsiella* vaccines are in development, targeting surface antigens; importantly, our analysis suggests that these surface antigens may be different in Malawi with implications for choice of vaccine targets in a setting where the *K. pneumoniae* species complex is a major cause of infant mortality. Finally, we show similar surface antigens in *K. pneumoniae* species complex isolates that are carried in stool and those causing disease in Blantyre, meaning that sequencing of colonizing isolates from stool could be a rapid and cost-effective way to map global *Klebsiella* surface antigen distributions to inform vaccine design.

## Data Summary

All data and code to replicate this analysis are available as the *blantyreESBL* v1.1.4 R package (https://doi.org/10.5281/zenodo.5554081) available at https://github.com/joelewis101/blantyreESBL. Reads from all isolates sequenced as part of this study have been deposited in the European Nucleotide Archive, and sample accession numbers (as well as accession numbers of publicly available genomes used in this analysis) are provided as supplementary data and in the R package.

## Introduction

*Klebsiella pneumoniae* is a highly prevalent human gut colonizer [1], opportunistic pathogen [2] and a major cause of neonatal sepsis worldwide [3]. It is often associated with antimicrobial resistance (AMR) and has been identified by the World Health Organization as a global priority AMR pathogen [4]. In low- and middle-income countries (LMIC) such as the nations of sub-Saharan Africa, multidrug-resistant *K. pneumoniae,* especially strains producing extended-spectrum beta lactamase (ESBL) enzymes present a significant therapeutic challenge. In many countries in sub-Saharan Africa, third-generation cephalosporin (3GC) antimicrobials are the mainstay of management of severe febrile illness [5–7], but ESBL production renders these antimicrobials ineffective. For example, in Malawi, a low-income country in South-East Africa, 91 % of *K. pneumoniae* infections are now resistant to third-generation cephalosporins at one major centre [8]; the BARNARDS study in seven LMIC found *K. pneumoniae* to be the commonest cause of neonatal sepsis, with all isolates resistant to 3GC [9]. In Malawi, as many other LMIC, alternatives to 3GC with activity against ESBL-producers such as carbapenems are often unavailable, rendering ESBL *K. pneumoniae* infections de facto untreatable with locally available antimicrobials.

Whole-genome sequencing (WGS) has provided significant insight into the population structure of *K. pneumoniae *; we now understand that isolates phenotypically identified as *K. pneumoniae* belong to a species complex encompassing several subspecies [10] – the *K. pneumoniae* species complex (KpSC). WGS has highlighted that the global spread of MDR KpSC is linked to the spread of resistance-encoding mobile elements and clonal expansion of MDR-associated high-risk clones [2]. Furthermore, genomic loci associated with virulence [11] (in particular the hypermucoid phenotype [12]) have been identified. Historically, AMR and virulence were associated with distinct populations, but AMR genes in hypervirulent lineages are increasingly observed, especially in South and South-East Asia [13]. These may result in community-acquired widely disseminated infections in otherwise healthy individuals that are difficult to treat [14].

In response to this urgent threat to public health, *K. pneumoniae* vaccines are in development [15]. Surface-exposed polysaccharides are an attractive vaccine target, particularly LPS O-antigens, which can be predicted via a sequence-based typing scheme [16, 17]. Analyses of large-scale genome collections have provided important insights into the distribution and diversity of the species complex, which is essential to focus efforts to the clinically most relevant types [16, 18]. An initial global collection [10] represented a milestone in KpSC genomics; it provided our first insight into the genomic plasticity, but it was restricted to isolates from 12 countries, notably lacking any sub-Saharan Africa representatives. Follow-up studies over the past years have also mainly focused on high-income country (HIC) clinical studies [19, 20], and genomes from LMICs, in particular sub-Saharan Africa, are drastically underrepresented in genome datasets. There is an urgent need to investigate the genomic epidemiology of the KpSC in this setting, where these pathogens are responsible for so much neonatal mortality [9], and to assess whether conclusions from largely HIC collections are valid for LMICs. This is a crucial requirement for a vaccine to be effective in these settings where, it is likely to have the most benefit.

In addition, though colonization with members of the KpSC is thought to precede infection in many cases [1], sequencing efforts have focused mainly on invasive isolates. There is some evidence from elsewhere that colonizing and invasive isolates differ [2]; understanding this difference in sub-Saharan Africa could help to define the determinants of infection in this setting. We, therefore, present the results of a genomic analysis of KpSC genomes, which were sequenced for a study of colonisation with ESBL Enterobacterales in Blantyre, Malawi. The results of that study are presented elsewhere [21], but the analysis here has three aims: (i) to describe the population structure, serotype diversity, AMR and virulence determinants of colonising KpSC isolates in this setting; (ii) to compare colonizing to previously sequenced Malawian invasive isolates, and (iii) to relate these data to observations made in other parts of the world.

## Methods

The isolates analysed in this study were colonizing isolates selectively cultured from stool and/or rectal swabs collected from adults in Blantyre, Malawi, as part of a study of longitudinal carriage of ESBL-producing Enterobacterales, as previously described [21]. Briefly, we recruited three groups of adults (≥16 years): (i) 225 adults with sepsis in the emergency department of Queen Elizabeth Central Hospital (QECH), Blantyre, Malawi, (ii) 100 antimicrobial-unexposed adults admitted to QECH and (iii) 100 antimicrobial-unexposed community dwelling adults, who lived within 30 km of Blantyre city. Queen Elizabeth Central Hospital is a tertiary referral, centre and the only government hospital providing free care in Blantyre (population 800064 in the 2018 census [22]). Antimicrobial-unexposed was defined as with no receipt of antimicrobials in the previous 4 weeks except for long-term co-trimoxazole preventative therapy (CPT, trimethoprim-sulfamethoxazole administered lifelong to people living with HIV in Malawi as per World Health Organization [WHO] guidelines [23]) or antituberculous chemotherapy, (usually comprising of rifampicin, isoniazid, pyrazinamide and ethambutol). Up to five stool samples per participant (or rectal swab samples performed by trained study team members if participants were unable to provide stool) were collected over 6 months. These were aerobically cultured overnight at 37 °C on ChromAGAR ESBL-selective chromogenic media (ChromAGAR, France) before being speciated with the API system (Biomeriuex, France). Full longitudinal carriage data are presented elsewhere [21] but 1416 stool samples were collected from the 425 participants; isolates phenotypically identified as ESBL *K. pneumoniae* were cultured from 233 samples.

DNA was extracted from the first 217/233 isolates (this number was set by logistic constraints). DNA was extracted from overnight nutrient broth cultures using the Qiagen DNA Mini kit (Qiagen, Germany) as per the manufacturer’s instructions. DNA was shipped to the Wellcome Sanger Institute for paired-end 150 bp sequencing on the Illumina HiSeq X10 instrument. Species was confirmed with Kraken v1.1.1 and Bracken v2.5 (with a 8 Gb MiniKraken database constructed on 3 April 2018) [24]. *De novo* assembly was undertaken with SPAdes v3.10.0 [25] followed by the assembly pipeline by Page *et al*. [26] and the quality of the assemblies assessed with QUAST v5.0.2 [27] and CheckM v1.1.2 [28]; assembly failures with a total assembled length of <4 Mb or assemblies with a CheckM-defined contamination of ≥10 % were excluded from further analysis. Overall, 203 genomes passed QC and were analysed further. Assemblies were then annotated with Prokka v1.14.5 with a genus-specific database from RefSeq [29] and the Roary v3.13 pangenome pipeline [30] used to identify core genes with default settings and paralogues not split. Genes present in ≥99 % samples were considered to be core. In total, 20853 genes were identified, of which 3391 were core. These were concatenated to a 2.82 Mb pseudosequence; the 378596 variable sites were extracted with SNP-sites v2.5.1 [31] and used to infer a maximum-likelihood phylogeny with IQ-TREE v1.6.3 [32], with 1000 ultrafast bootstrap replicates. The IQ-TREE ModelFinder module was used to select the best fitting nucleotide substitution model, using the *-fconst* option to account for nonvariable sites; this selected a general time reversible model with FreeRate site heterogeneity and eight parameters. Trees were visualised using the R *ggtree* v2.2.4 [33] package.

Kleborate v2.0.1 [34] with default settings was used to infer *Klebsiella* species, capsule polysaccharide (K-type) and lipopolysaccharide (O-type) serotypes, and to identify the presence of the siderophore virulence loci *ybt* (yersiniabactin), *iuc* (aerobactin) and *iro* (salmochelin)*,* the genotoxin locus *clb* (colibactin)*,* and the hypermucoidy genes *rmpA* and *rmpA2,* using default settings. K- and O-types were recoded as ‘unknown’ if the Kleborate-defined confidence in their identification was below ‘good’. Kleborate identifies the genes most robustly associated with *Klebsiella* virulence [34] (including those associated with hypervirulence) but in addition we used ARIBA v2.14.6 [35] with default settings to screen for other virulence genes from the VFDB-core database [36]. ARIBA (again with default settings) was used to determine multilocus sequence type (ST) as defined by the 7-gene Pasteur scheme [37] hosted at pubMLST (https://pubmlst.org/), to identify AMR-associated genes using the SRST2 curated version of the ARG-ANNOT database [38] and to call SNPs in the quinolone-resistance determining regions (QRDR) *gyrA, gyrB, parC* and *parE*, using the wild-type genes from the *Escherichia coli* K-12 substr. MG1655 (NC_000913.3) as reference. Quinolone resistance was assumed to be conferred by QRDR mutations recorded in the Comprehensive Antibiotic Resistance Database [39] (CARD) as causing quinolone resistance in Enterobacterales. Beta-lactamases were considered to be extended spectrum based on the phenotypic classifications at https://ftp.ncbi.nlm.nih.gov/pathogen/betalactamases/. We explored clustering of AMR genes using hierarchical clustered heatmaps of Jaccard distances of AMR gene presence using the base *dist* and *hclust* functions in R, visualized with the pheatmap package v1.0.2.

Two further phylogenies were constructed to place the isolates from this study in a local and global phylogenetic context. Firstly using collections of Malawian isolates [40, 41], and secondly from a multi-country, large-scale description of KpSC population structure [10] that was designed to span the diversity of the species with samples collected between 1973 and 2011. This was combined with 66 genomes from a study of the genomic epidemiology of *K. pneumoniae* in Kenya [42]. Two studies from QECH provided the previously published Malawian genomes. The first was a genomic investigation into a *K. pneumoniae* outbreak on the neonatal ward at QECH [40], which sequenced 100 bloodstream infection isolates from children between 2012 and 2015. The second was a study, which sequenced 72 sterile-site (blood and CSF) and rectal colonizing isolates selected to maximize diversity [41] from QECH from 1996 to 2014. The same quality control steps, assembly, annotation, ST and AMR gene identification and pangenome determination steps were applied; following QC, 150 genomes from samples collected in Malawi were combined with the 203 from this study. Details of the studies from which contextual isolates are drawn are shown in Table 1, along with the proportion of invasive versus colonizing isolates and ESBL isolates. We defined invasive isolates as those cultured from normally sterile sites and colonizing isolates those cultured from stool or rectal swab. The exception being if studies providing contextual isolates themselves defined invasive/colonising. If this was the case we accepted the study definition. Because the Cornick *et al*. study isolates [40] were from an outbreak investigation, which identified a putative ST340 outbreak lineage on the neonatal ward we assessed diversity in ST from this study. We plotted the ST distribution and calculated the Shannon diversity index with bootstrapped 95 % confidence intervals using the *vegan* v2.5 package and 1000 bootstrap replicates (Fig. S1 and Table 1). Despite the fact that the outbreak investigation identified 17 isolates from the putative outbreak lineage, ST diversity was similar between the two Malawian studies including invasive isolates, and slightly greater in the isolates sequenced for this study. We therefore included all Cornick *et al*. isolates in further analysis.

**Table 1.** Details of studies included in contextual analysis

Study	Country/ies of isolate collection (proportion)	Original inclusion criteria	Number of isolates in original publication	Number of isolates included following QC	Number (proportion) invasive isolates	Number (proportion) ESBL isolates	Shannon diversity in ST* (95 % CI)
This study	Malawi (1.00)	See Methods	217	203	0/203 (0.00)	200/203 (0.99)	3.57 95 % CI (3.45–3.70)
Cornick *et al* 2021 [40]	Malawi (1.00)	All BSI isolated from neonatal wards QECH 2012–2015 (*n*=62); sample of isolates from paediatric wards QECH 2012–2015 (*n*=38)	100	79	79/79 (1.00)	66/79 (0.84)	2.71 95 % CI (2.43–2.95)
Musicha *et al* [41]	Malawi (1.00)	Samples selected from stored isolates from routine bacteraemia and meningitis surveillance QECH from adults and children 1996–2014 (*n*=59) Rectal swabs from Klebsiella carriage survey QECH 2009 (*n*=13)	72	71	60/71*** (0.85)	40/71 (0.56)	3.17 95 % CI (2.89–3.40)
Holt *et al* [10]	USA (0.35), Vietnam (0.27), Australia (0.14), Laos(0.09), Indonesia (0.07), Singapore (0.06), UK (<0.01)	Stored isolates selected to maximise diversity	288	264	142/187 (0.76)	104/264 (0.39)	–
Henson *et al* 2017 [42]	Kenya (1.00)	Klebsiella BSI from Kilifi County Hospital 1994–2007	66**	65	65/65 (1.00)	43/65 (0.66)	–

Notes: *Calculated for Malawian studies only.

**We included the same 66 Kenyan isolates selected as context using the methods described in Musicha *et al.*; the original publication included 198 isolates.

***Of the 60 invasive isolates from Musicha *et al*., 57 were from blood and three from CSF.

Invasive versus colonizing defined using original study definitions, or, if not available invasive is defined as an isolate from a sterile site and colonizing as an isolate from stool or rectal swab.

BSI, Bloodstream infection; QECH, Queen Elizabeth Central Hospital, Blantyre, Malawi; ST, Sequence type.

Roary identified a pangenome in this 353-genome Malawian collection of 20853 genes of which 3 391 were core. These core genes formed a 2.82 Mb alignment with 378596 variable sites, which were used to infer a phylogeny as described above, using the same nucleotide substitution model. To build a global phylogeny we included all Malawian isolates plus the 66 genomes from Kenya [42] and the 288 genomes from the Holt *et al*. multi-country [10] collection, again using the same methods. In total, following QC, this analysis included 687 genomes; the pan-genome as constructed with Roary comprised 49385 genes, of which 2754 were core; these formed a concatenated pseudosequence of 0.95 Mb with 200622 variable sites, which were used to infer a phylogeny as above; ST and ESBL presence or absence were also inferred as described above.

All statistical analyses were carried out in R v4.1.1 (R Foundation for Statistical Computing, Vienna, Austria). Summary statistics, where presented, are medians and interquartile ranges or proportions with exact binomial confidence intervals unless otherwise stated. Comparisons of proportions use Fisher’s exact test; to compare proportions of each O- and K- type between invasive and colonizing isolates, we corrected for multiple comparisons using the Benjamini-Hochberg *P*-value correction. *P*<0.05 was considered statistically significant. The clinical study, which provided the isolates for this analysis, was approved by the Liverpool School of Tropical Medicine (16-062) and Malawi College of Medicine (P.11/16/2063) research ethics committees. All sequenced isolates have been deposited in the European Nucleotide Archive, and accession numbers (as well as accession numbers of publicly available genomes used in this analysis) and metadata are provided as supplementary data (Fig. S1) and in the R package linked to this publication: the *blantyreESBL* R package [43] available at https://joelewis101.github.io/blantyreESBL/. This package also includes all code and data to reproduce the analyses in this manuscript.

## Sensitivity analyses

To assess the effect of putative biases introduced by our sampling scheme on our analyses comparing invasive to colonizing Malawian isolates we performed additional sensitivity analyses. The possible sources of bias we identified were as follows: (1) bias introduced by selecting only ESBL-producing colonising isolates for sequencing; (2) bias introduced by using multiple samples from within a single participant that may represent repeat sampling of the same clone; and (3) bias introduced by including isolates from the Malawian outbreak investigation. To account for these potential sources of bias we defined a sensitivity analysis population, which included all Malawian samples as described but (1) excluded samples without a detectible ESBL gene (*n*=44/353); (2) in cases where the same individual carried the same ST at multiple time points, we randomly selected one sample only to include and excluded the rest (excluding *n*=19/353 samples); and (3) excluding all ST340 isolates (the putative outbreak strain, *n*=18/353) from the Cornick *et al*. outbreak investigation. In total the sensitivity analysis population included 269 samples. We compared the ST, K- and O-type distribution in the sensitivity analysis population to the full sample collection and used the sensitivity analysis population to repeat the analyses comparing virulence determinants and surface antigens of invasive and colonising isolates, as above.

## Results

### Population structure

Following QC, 203 KpSC genomes (of the 217 sequenced) were included in our analysis, from 142 participants, a median of 1 (1-2) isolates per participant: 154 isolates from 100 participants with sepsis; 34 from 26 antimicrobial unexposed inpatients and 16 from 15 community members. Participant characteristics and a full analysis of within-participant diversity are presented elsewhere [21], but where participants contributed multiple samples to the analysis, they were usually of different STs: 48 participants contributed more than one sample to the analysis, 109 samples in total. Of these 72/109 were assigned an ST that was not isolated more than once from a given participant. Of the remaining samples, one ST was represented twice in one participant 17 times, and three times in one participant once.

**Fig. 1.**
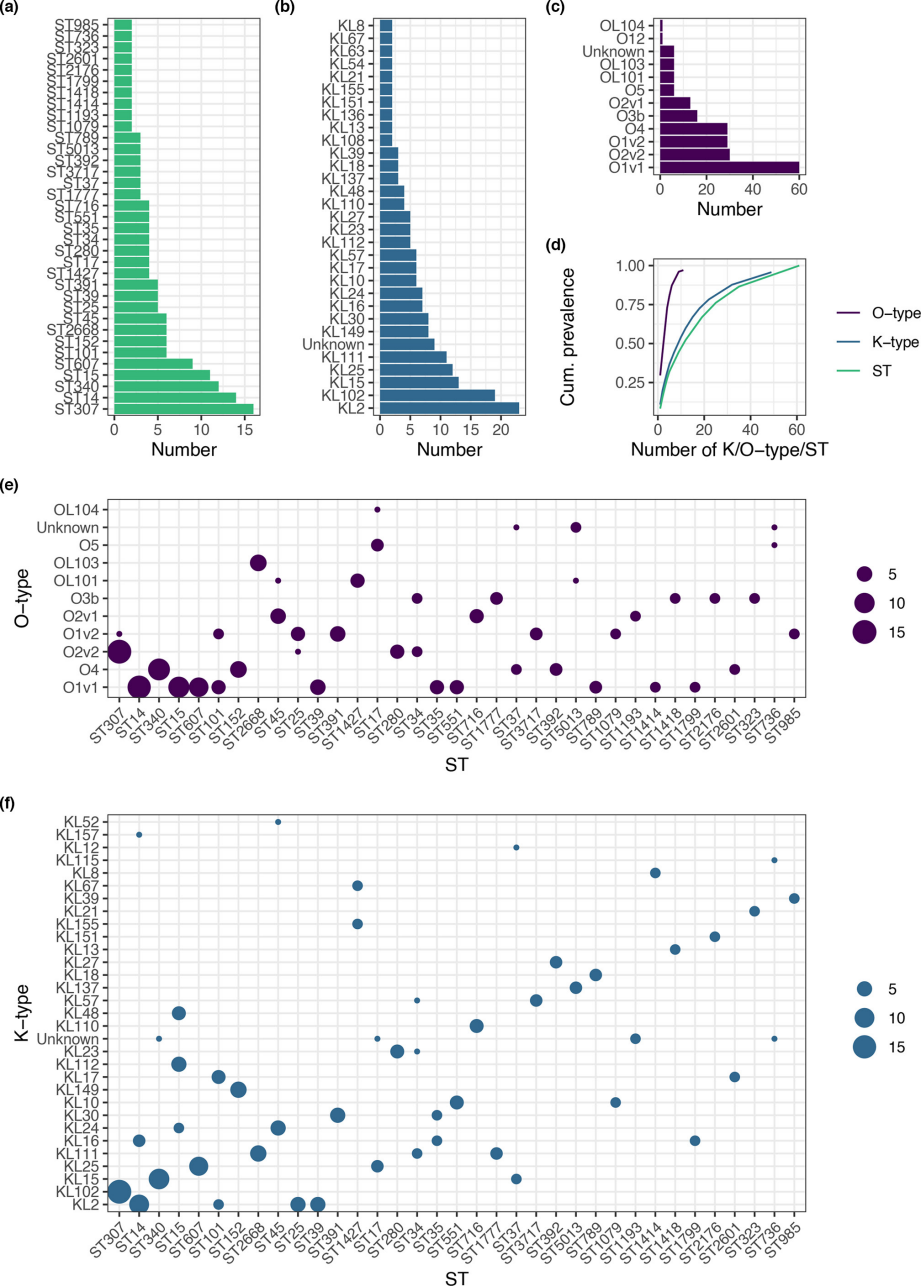
Diversity of the 203 colonizing *K. pneumoniae* sequence complex genomes sequenced for this study. Distributions of (a) sequence type (ST), (b) K-type, (c) O-type. (d) shows cumulative prevalence as a function of number of K/O-types or ST, where K/O-type or ST is ordered from largest to smallest. (e) and (f) show ST association of O-type and K-type, respectively, where the area of point is proportional to the number of samples. STs with only a single representative in the collection are excluded from plots (a), (b), (c), (e) and (f) .

Most of the 203 genomes were *K. pneumoniae* subsp. *pneumoniae* (*n*=190). *K. quasipneumoniae* subsp. *similipneumoniae* (*n*=7)*, K. variicola subsp. *variicola** (*n*=3), *K. quasipneumoniae* subsp. *quasipneumoniae* (*n*=2) and *K. quasivariicola* (*n*=1) were also represented, which is comparable to other clinical studies on the KpSC [10, 44–46]. Considering the number of samples, we identified a large number of STs (Fig. 1) with 61 identified and a median of 2 (IQR 1–4) isolates per ST; 26/61 STs were only represented once in the collection. The most common STs identified were ST307 (*n*=16), ST14 (*n*=14), ST340 (*n*=12) and ST15 (*n*=11). The STs reflected the structure of the inferred core-gene phylogeny well (Fig. S2), which, as expected, had the characteristic KpSC deep branching topology [2].

**Fig. 2.**
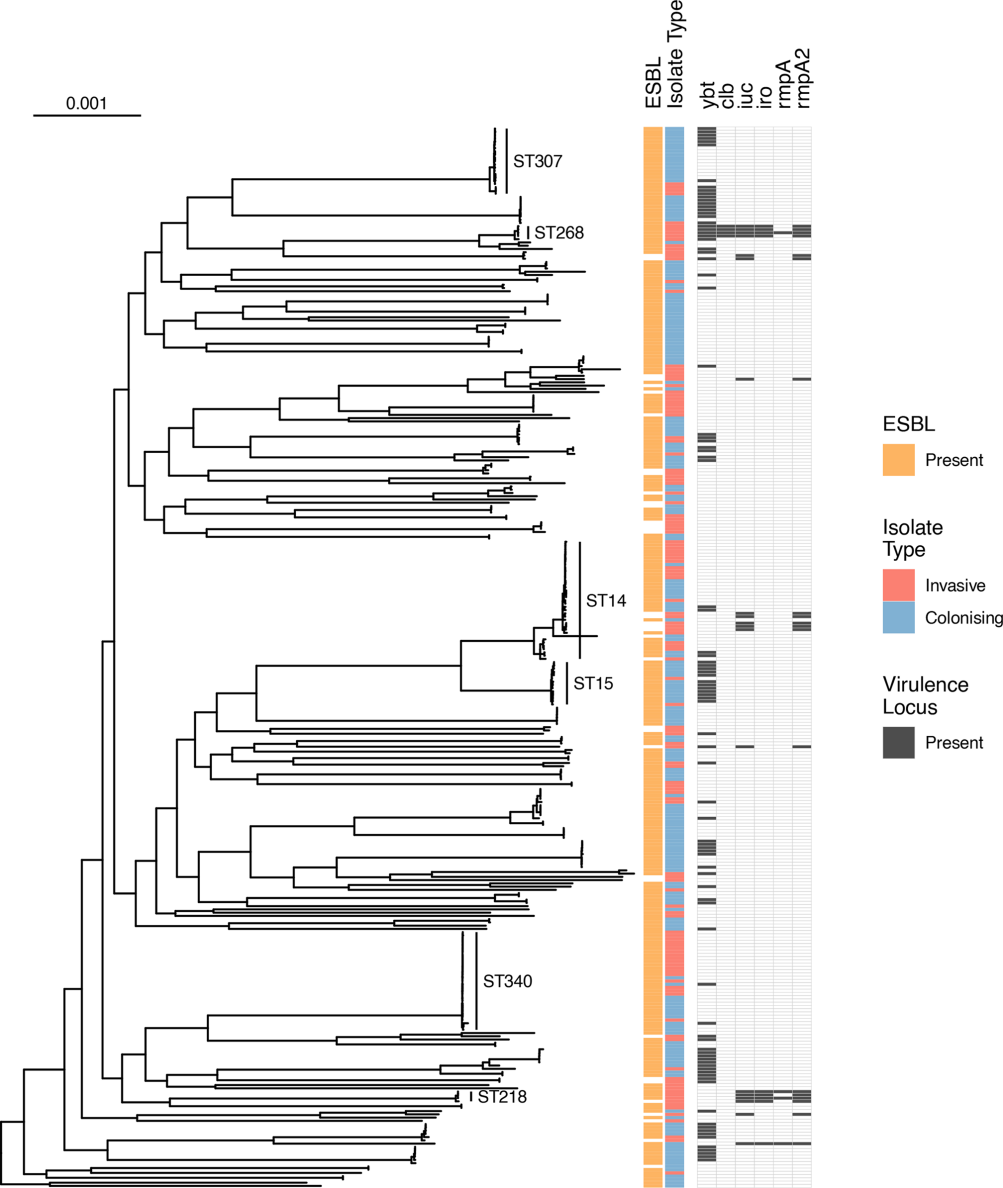
Midpoint rooted core-gene maximum-likelihood phylogenetic tree of Malawian isolates, including all genomes from this study and context genomes from Malawian studies (*n*=353), and restricted to *K. pneumoniae* subsp. *pneumoniae *. Heatmaps show whether ESBL genes are present, whether colonizing or infection, and whether the siderophore virulence loci *ybt* (yersiniabactin), *iuc* (aerobactin), *iro* (salmochelin), the genotoxin virulence locus *clb* (colibactin) and the hypermucoidy genes *rmpA* and *rmpA2* are present. Scale bar shows nucleotide substitutions per site.

**Fig. 3.**
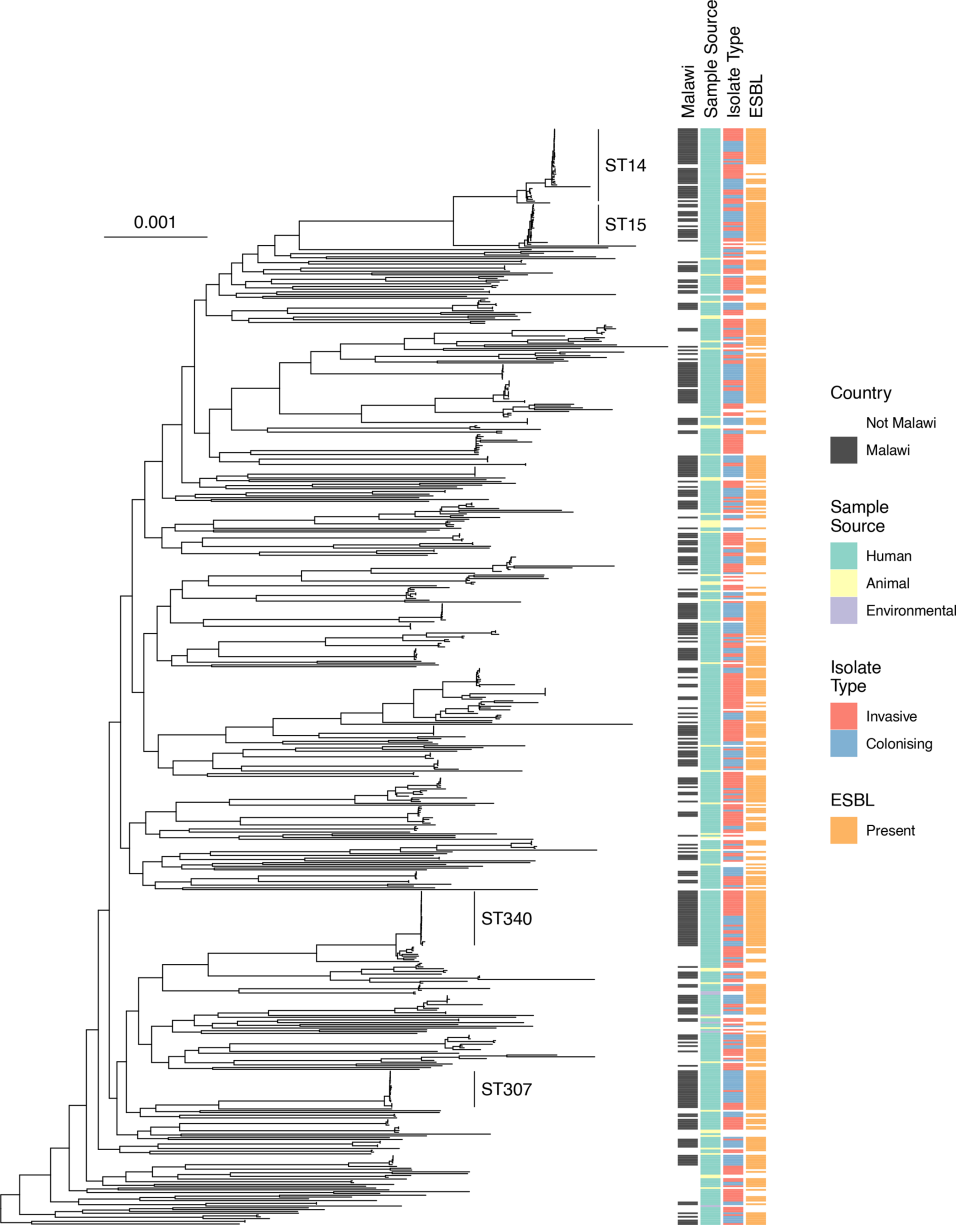
Midpoint rooted core-gene phylogenetic tree of Malawian and global isolates, restricted to *K. pneumoniae* subsp *. pneumoniae *. Heatmaps show whether isolated in Malawi, whether animal/human/environmental, whether colonising or infection, and whether ESBL genes are present. Scale bar shows nucleotide substitutions per site.

Incorporating previously sequenced invasive KpSC genomes isolated in Malawi from blood (*n*=136), cerebrospinal fluid (*n*=3) and colonizing isolates from rectal swab (*n*=11) to the phylogeny of the 203 colonizing isolates from this study – 353 genomes in total – revealed a similar population structure in *K. pneumoniae* subsp. *pneumoniae* between colonizing and invasive isolates (Fig. 2). The global core-gene-based phylogeny including multi-country context genomes and Malawian invasive and colonizing isolates (*n*=687) showed that the included Malawian isolates were distributed throughout the *K. pneumoniae* subsp. *pneumoniae* tree (Fig. 3) and mirrored the broader population structure of the included samples. The ST14 and ST15 isolates clustered with genomes from elsewhere but ST340 and ST307 were consistent with local clonal expansion.

### Antigenic and AMR gene diversity of Malawian colonizing isolates

In the 203 colonizing isolates sequenced for this study, 49 K-types were identified and were ST-associated with median 1 (IQR 1–2) STs per K-type. Eight O-types were identified: six of the 12 originally described O-types based on serology (O1 [v1 and v2], O2 [v1 and v2], 3b, 4, 5, 12), as well as three *rfb* loci predicted to encode novel O-types, which have been reported but not yet described serologically [47], denoted by OL: OL101, 103 and 104, though these were in the minority (13/203; Fig. 1, Fig. S2). O-types were more likely to be encoded by multiple STs than K types: each O-type was associated with a median of six (IQR 2.5–11.8) STs. The four most common predicted O-antigens were O1 (89/203 [44 %]), O2 (43/203 [21 %]), O4 (29/203 [14 %]) and O3b (16/203 [8 %]) accounting for 87 % of samples (Table S1). In contrast, the four most frequent K-types (K2 [11 %], 102 [9 %], 15 [6 %] and 25 [6 %], Table S2) and STs (ST307 [8%], 14 [7 %], 340 [6 %], 15 [5 %]) together accounted for only 33 and 26 % of samples, respectively.

**Fig. 4.**
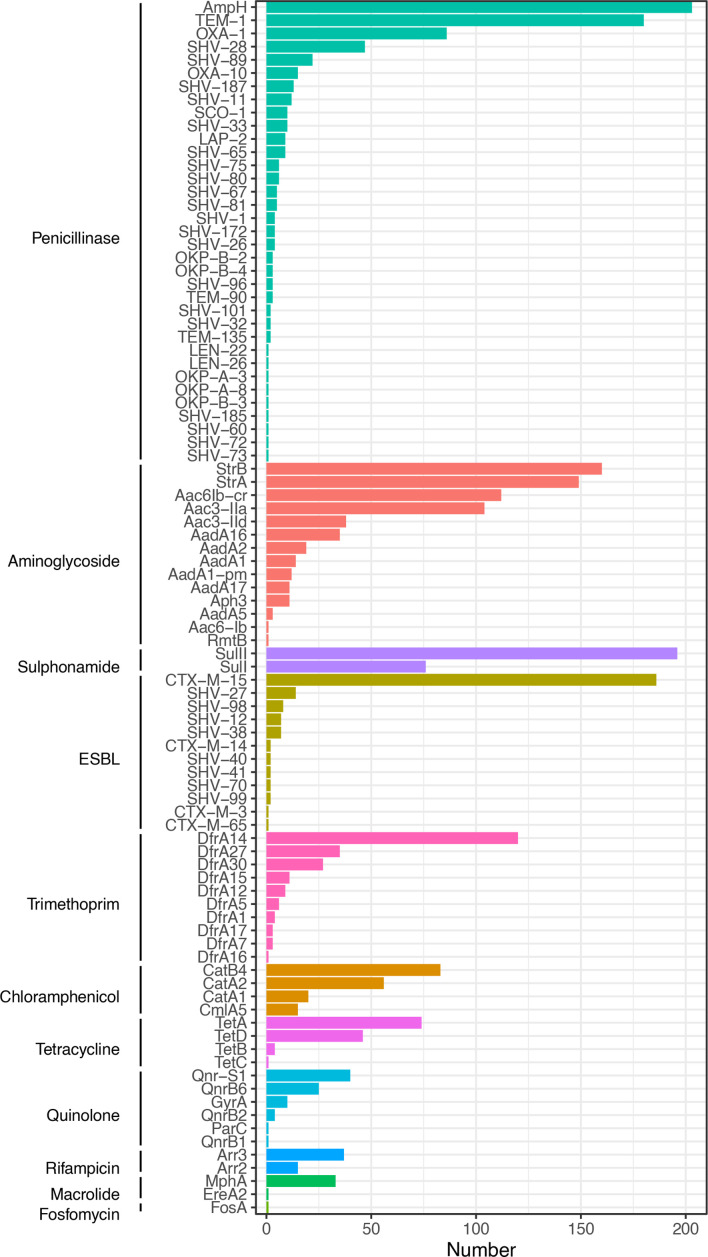
Antimicrobial resistance determinants identified.

The isolates contained a median of 15 (IQR 12–17, range 6–25) antimicrobial resistance genes (including QRDR SNPs) per genome (Fig. 4) consistent with previous studies of resistant KpSC isolates [44]. Consistent with isolates being grown on ESBL selective media, at least one ESBL-encoding gene was identified in 99 % of genomes (200/203). Genes encoding narrow spectrum beta-lactamases were also common (200/203, 99 % genomes, excluding the genus-associated *bla*_ampH_ penicillinase, which was present in 100 % of isolates). The most commonly identified ESBL-encoding gene was *bla*_CTX-M-15_ in 186/203 (92 %) of genomes. Genes conferring resistance to sulphonamides (201/203, 99 %), trimethoprim (198/203, 98 %) and aminoglycosides (198/203 98 %) were near-ubiquitous. Determinants of resistance to chloramphenicol (140/203, 69 %) and fluoroquinolones (76/203, 37 %) were less common. Quinolone resistance determinant region mutations identified were S83F (*n*=10) in *gyrA* and S80I (*n*=1) in *parC*, but *qnrB* (*n*=30) and *qnrS* (*n*=40), the plasmid-mediated quinolone resistance genes [48] were also identified. No known genes conferring carbapenemase resistance were identified.

Some AMR genes clustered together (e.g. *strA* with *strB, bla*_CTX-M-15_ with *bla*_TEM-1_ and *sulII,* and *bla*_SHV-11_ with *aadA1-pm* and *bla*_OXA-10_, Fig. S3), and some of these AMR-gene clusters were lineage-associated (e.g. the *bla*_SHV-11_ *aadA1-pm bla*_OXA-10_ cluster with ST340, Fig. S4).

### Comparing Malawian colonizing and invasive isolates

**Fig. 5.**
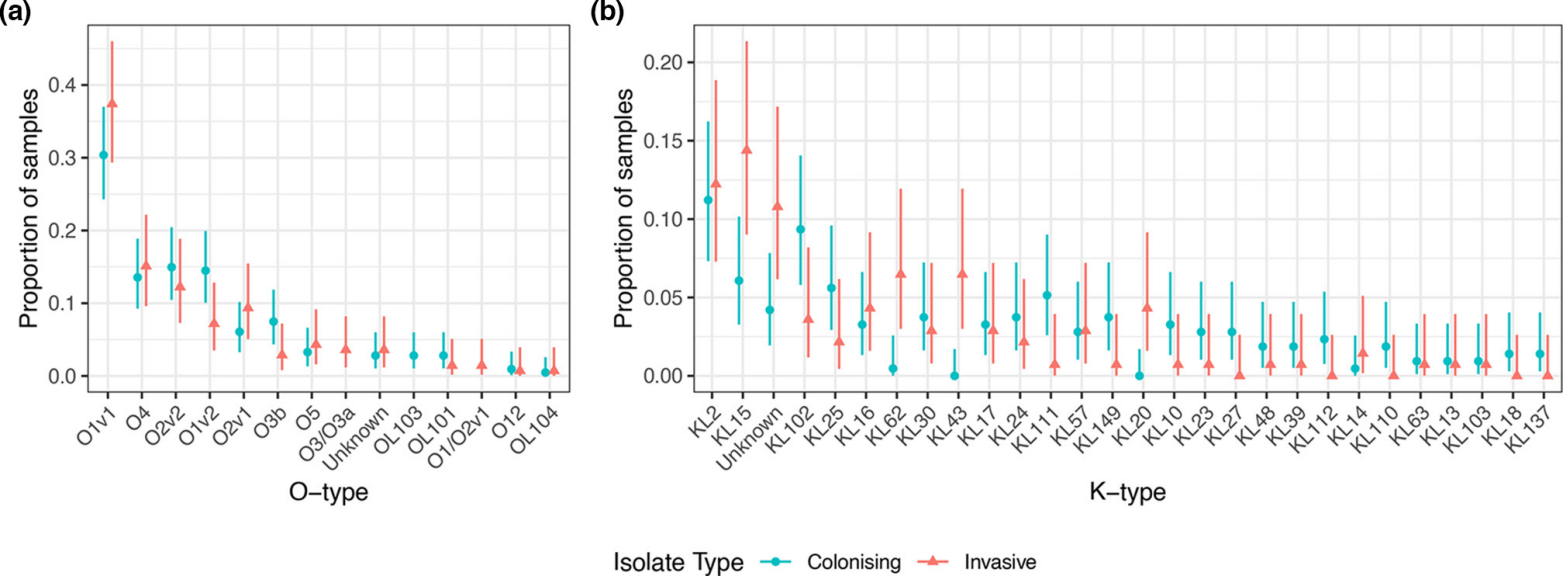
Distribution of O-types (a) and K-types (b) stratified by colonising or infecting samples, showing that the O-type distribution is similar whether infecting or colonizing.

Finally, we compared Malawian colonizing to invasive isolates based on K- and O-type and recognized virulence determinants using the collection of 353 Malawian invasive and colonizing genomes. O-type and K-type distributions were similar across invasive and colonizing isolates (Fig. 5). Fisher’s exact tests corrected for multiple comparisons were consistent with similar proportions of each O-type and K-type across infecting and colonizing isolates, except for KL62 (*P*=0.04) and KL43 (*P*=0.01, Tables S3 and S4). Both of these K-types were strongly associated with invasive isolates (9/10 KL62 and 9/9 KL43 were invasive) and associated with multiple STs: KL43 was present in ST372 (six isolates), ST106 (two isolates), ST276 (one isolate) and KL62 was present in ST664 (three isolates), ST48 (three isolates), ST348 (two isolates) and ST4 and ST432 (one isolate each). All of these isolates contained O-type 1 or 2.

The most commonly identified virulence determinant using Kleborate was the siderophore locus *ybt*, present in 27 % (94/353) of Malawian isolates, and more common in colonizing (68/214, 32 %) compared to invasive (26/139, 19 %, *P*=0.007) isolates, and in ESBL isolates (89/306, 29 %) compared to non-ESBL isolates (5/47, 11 %, *P*=0.007). All other virulence determinants were less common (the siderophore loci *iuc* in 5 % and *iro* in 3 %; the genotoxin loci *clb* in 1 %; and the hypermucoidy genes *rmpA2* and *rmpA* in 5 and 1%, respectively) but were associated with invasive isolates, except for *rmpA* (Table S5). Only 1/214 (0.5 %) colonizing isolate contained any of these virulence genes (Figs 2 and S5 and Table S5), compared to 18/139 of invasive isolates (12.9 %, *P*<0.001). Generally, isolates containing these non-*ybt* virulence determinants were less likely to contain ESBL-encoding genes (11/47 [23 %] non-ESBL isolates vs 8/306 [3 %], *P*<0.001) but two lineages contained both: ST268 (four isolates with *ybt, clb, iro,iuc* and *rmpA2* +/-*rmpA*) and ST218 (four isolates with *iuc, iro* and *rmpA2* +/-*rmpA*) also all carried ESBL-encoding genes. These ST218/268 isolates were all from blood culture, the earliest in 2004 and no isolates of these STs were cultured from colonising samples. All ST218 were identified as K-locus KL57 and ST268 as KL20; both have been described as virulence-associated K-types [13].

We additionally screened the collection against the Virulence Factor Database (VFDB), which did not identify any other virulence determinants in the collection that were associated with invasive isolates, but identified a core set of five virulence factors that were present in >95 % isolates. These included genes encoding type 1 and 3 fimbriae, type VI secretion systems, the AcrAB efflux pump, and the transcriptional capsule regulator *rcs*. There was also sporadic presence of other virulence factors (Table S6), though none were associated with invasive isolates. Genes encoding the *E. coli* common pilus were more common in colonizing (30/214 [14 %]) as compared to invasive isolates (2/139 [1 %], *P*<0.001); these were not lineage associated and were distributed through the phylogeny (Fig. S6)

The K- and O-type and ST distributions of the sensitivity analysis population were similar to the full sample set (Fig. S7) and antigenic and virulence comparisons of Malawian invasive versus colonizing isolates were largely unchanged in the sensitivity analysis population (Fig. S8 and Tables S5 and S6). This analysis demonstrated that some of the association of *ybt* with colonizing isolates was due to association with ESBL; in the sensitivity analysis population more colonizing than invasive isolates carried *ybt* but this was no longer statistically significant (21/78 [27 %] invasive vs 61/191 [32 %] colonizing, *P*=0.467). KL43 remained associated with invasive isolates (Benjamini-Hochberg corrected *P*-value <0.001) but KL62 was not (Benjamini-Hochberg corrected *P*-value 0.96); however seven of the ten isolates encoding KL62 were excluded from the sensitivity analysis population as they were did not carry ESBL genes.

## Discussion

Malawi, and sub-Saharan Africa in general, is an area of the world that is underrepresented in current *K. pneumoniae* collections. We present a genomic investigation of 203 colonizing ESBL-producing KpSC genomes from Blantyre. Placing these in both a Malawian and broader context enables us to draw several conclusions that increase our understanding of the dynamics of colonisation and infection in this setting, as well as understand the differential threats for the global emergence of drug resistant *Klebsiella* from all parts of the world.

The isolates causing infection in Malawi and the isolates from colonizing represent highly similar population structures, consistent with the hypothesis that the source of infecting KpSC is the host microbiota [1]. Importantly, the diversity seen in the Malawian ESBL *K. pneumoniae* subsp. *pneumoniae* reflects the diversity of *K. pneumoniae* subsp. *pneumoniae* isolates from multiple countries, suggesting that the samples we include from Malawi are sampling global diversity. However, there are important local differences: some STs are overrepresented in the Malawian isolates compared to the multi-country collections we used for context. These differences suggest different requirements for KpSC success as an invasive pathogen and subsequent clonal expansion in Malawi compared the high-income settings included in the global collection.

Successful Malawian STs (i.e. having undergone clonal expansion) included ST14 and ST15, which are common ESBL and carbapenemase-associated *K. pneumoniae* subsp. *pneumoniae* lineages particularly in Asia [13]. Malawian isolates clustered closely with global context ST14/15 genomes in the core gene tree. In comparison, the core gene tree topology of the common Malawian ST307 and ST340 lineages was consistent with local subclades. These apparent local subclades may be representative of the biases inherent in the sampling frame of the multi-country collection; ST307 and ST304 are common ESBL- and carbapenemase-associated lineages in North [19] and South [49] America, and in Europe [20], but were unusual in the earlier multi-country collection we used for context [10].

The drivers of local or regional success for some lineages over others are not clear; in Malawi, success was not explained by the identified virulence determinants. The well described virulence loci *clb, ioc, iuc* and hypermucoidy genes *rmpA* ad *rmpA2* are, as expected, present in multiple STs causing invasive disease. By comparing colonizing isolates sequenced for this study with previously sequenced invasive isolates, we found that, although presence of these loci was associated with invasive disease, the majority of invasive isolates lacked them. Host factors or other pathogen factors (e.g. capsule) may therefore be the primary determinant of whether colonizing develops into infection in the Malawian setting. Of note, the K-types KL62 and KL43 were associated with invasive disease in our setting. Generally, when considering all Malawian isolates (i.e. colonizing isolates, which were selected for ESBL production, and invasive isolates, which were not) presence of virulence determinants was associated with absence of ESBL-encoding genes, as described elsewhere in the world [2]. However, two lineages (ST218 and ST268) demonstrated the presence of both, indicating that hypervirulent-AMR Klebsiella lineages are present in Malawi.

AMR-gene content was diverse in Malawian colonizing isolates and *bla*_CTX-M-15_ was by far the most commonly identified ESBL-encoding gene, present in 92 % of isolates. Presence of trimethoprim and sulphonamide resistance determinants was near-universal. In Malawi, a high-HIV prevalence setting, the WHO recommends lifelong co-trimoxazole preventative therapy (CPT) for people living with HIV [23], and wide availability means it is frequently used as a mainstay of community antimicrobial chemotherapy in health centres in Malawi (source: Ministry of Health Malawi). This raises the possibility that strains containing ESBL and co-trimoxazole resistance determinants could be selected for by CPT in the absence of beta-lactam use and this combination of AMR genes is typical in this collection. It may be that a more nuanced approach to CPT is necessary in an era of increasing Gram-negative resistance. No genes conferring resistance to carbapenemases were identified. Carbapenem resistance is unusual in Malawi, though the carbapenemase *bla*_NDM-5_ has been described in *E. coli* in Blantyre contemporaneously with this study [50], and the carbapenemases *bla*_KPC-2_ in ST340 *K. pneumoniae* and *bla*_OXA-48_ in *K. variicola* have been described in the central region of Malawi [51] in 2016/17. Carbapenems are increasingly available in QECH and it seems very likely that increasing exposure will result in rapid expansion of carbapenemases in *K. pneumoniae* especially given the likely unrestricted global flow of KpSC strains suggested by our data.

There was significant diversity in the Malawian collection in K- and O-types, as is characteristic of KpSC collections [13, 18, 52]. Vaccines based on KpSC O antigens are in development [15], and quadrivalent vaccines with various O-antigen targets have been proposed, depending on the cohort used to describe O-antigen epidemiology. In one global collection of 645 isolates phenotypically identified as *K. pneumoniae* [18], a quadrivalent conjugate vaccine based on antigens O1, 2, 3 and 5 might be expected to have activity against 90 % of isolates in the collection. A second study suggested O2v2, O1v1, O3b, O1v2 would cover 71–77 % of European isolates, based on a derivation isolate collection from Oxfordshire, UK [52]. O-antigen variation is apparent depending on location of isolation: the global collection described above [18] contained 11 % O5 containing isolates, but the Oxfordshire collection [52] 3 %, as did our collection. In the Malawian setting, we identified O4 in 14 % of colonizing and 15 % of infecting KpSC isolates, but this was rare in the multi-country (2 %) [18] and Oxfordshire (4 %) [52] collections and also in South East Asia [13] (2–4 %). These differences highlight the need for longitudinal surveillance and truly global collections, describing secular trends in O- and K- antigen epidemiology from diverse settings to guide vaccine development. We found that the O-type distribution for Malawian ESBL-producing KpSC colonizing isolates was similar to invasive isolates. This suggests that stool or rectal swab sampling with selective culture could be a cost-effective way to rapidly expand understanding of worldwide O-type distributions to guide vaccine development. This finding must be confirmed in further sites before adopting such a strategy.

There are limitations to our study. Most importantly, our sampling scheme is not unbiased: ESBL-producing colonizing isolates were selected for, because of the focus on ESBL-producers in the colonization study from which these samples arise [21]. This focus is because of the significant clinical difficulties ESBL-producing bacteria present in our setting. In addition, one of the Malawian studies providing invasive context genomes was an investigation of a *K. pneumoniae* outbreak on the QECH neonatal unit, and both of these sample selection strategies are likely to have introduced bias into the collection of Malawian genomes, especially against classically hypervirulent but antimicrobial susceptible lineages. Multiple samples were cultured from single individuals and so were not independent, which could introduce bias, however most individuals were colonized by different strains at different time points [21]. Of note, our findings were unchanged following sensitivity analysis. All Malawian genomes are from a single centre, which enables us to compare the population structure of colonizing and clinical isolates but may limit generalization to other settings in Malawi, or sub-Saharan Africa.

In conclusion, we present a genomic analysis of ESBL KpSC colonizing adults in Blantyre, Malawi. Malawian colonizing and invasive isolates are similar and population structure is comparable to our broader understanding of the KpSC population structure, suggesting that Malawi is sampling global *Klebsiella* diversity. However, we demonstrate that some lineages (ST14, ST15, ST307, ST340) are successful in the Malawian setting and have undergone expansion in this setting. The reason for this success is not explained by the virulence factors we sought and host factors, pathogen opportunity, or alternate virulence factors not linked to disease elsewhere may be the major determinants of lineage success and invasive disease in Malawi. O-antigen distributions of Malawian isolates are different to previously described collections, highlighting the need for geographically aware surveillance to inform vaccine development. Predicted O-antigen diversity was similar across invasive and colonizing isolates, suggesting that O-typing of colonizing isolates could be a cost-effective way to rapidly carry out such surveillance and assess putative O-antigen vaccine coverage across diverse populations.

## Supplementary Data

Supplementary material 1

Supplementary material 2

## References

[1] Gorrie CL, Mirceta M, Wick RR, Edwards DJ, Thomson NR (2017). Gastrointestinal carriage is a major reservoir of *Klebsiella pneumoniae* Infection in intensive care patients. Clin Infect Dis.

[2] Wyres KL, Lam MMC, Holt KE (2020). Population genomics of *Klebsiella pneumoniae*. Nat Rev Microbiol.

[3] Okomo U, Akpalu ENK, Le Doare K, Roca A, Cousens S (2019). Aetiology of invasive bacterial infection and antimicrobial resistance in neonates in sub-Saharan Africa: a systematic review and meta-analysis in line with the STROBE-NI reporting guidelines. Lancet Infect Dis.

[4] World Health Organisation (2017). Prioritization of Pathogens to Guide Discovery, Research and Development of New Antibiotics for Drug-Resistant Bacterial Infections, Including Tuberculosis.

[5] Lester R, Haigh K, Wood A, MacPherson EE, Maheswaran H (2020). Sustained reduction in third-generation cephalosporin usage in adult inpatients following introduction of an antimicrobial Stewardship Program in a Large, Urban Hospital in Malawi. Clin Infect Dis.

[6] Labi A-K, Obeng-Nkrumah N, Dayie NTKD, Egyir B, Sampane-Donkor E (2021). Antimicrobial use in hospitalized patients: a multicentre point prevalence survey across seven hospitals in Ghana. JAC Antimicrob Resist.

[7] Horumpende PG, Mshana SE, Mouw EF, Mmbaga BT, Chilongola JO (2020). Point prevalence survey of antimicrobial use in three hospitals in North-Eastern Tanzania. Antimicrob Resist Infect Control.

[8] Musicha P, Cornick JE, Bar-Zeev N, French N, Masesa C (2017). Trends in antimicrobial resistance in bloodstream infection isolates at a large urban hospital in Malawi (1998–2016): a surveillance study. Lancet Infect Dis.

[9] Sands K, Carvalho MJ, Portal E, Thomson K, Dyer C (2021). Characterization of antimicrobial-resistant Gram-negative bacteria that cause neonatal sepsis in seven low- and middle-income countries. Nat Microbiol.

[10] Holt KE, Wertheim H, Zadoks RN, Baker S, Whitehouse CA (2015). Genomic analysis of diversity, population structure, virulence, and antimicrobial resistance in *Klebsiella pneumoniae*, an urgent threat to public health. Proc Natl Acad Sci U S A.

[11] Lam MMC, Wick RR, Wyres KL, Gorrie CL, Judd LM (2018). Genetic diversity, mobilisation and spread of the yersiniabactin-encoding mobile element ICEKp in *Klebsiella pneumoniae* populations. Microb Genom.

[12] Walker KA, Miner TA, Palacios M, Trzilova D, Frederick DR (2019). A *Klebsiella pneumoniae* regulatory mutant has reduced capsule expression but retains hypermucoviscosity. mBio.

[13] Wyres KL, Nguyen TNT, Lam MMC, Judd LM, van Vinh Chau N (2020). Genomic surveillance for hypervirulence and multi-drug resistance in invasive *Klebsiella pneumoniae* from South and Southeast Asia. Genome Med.

[14] Russo TA, Marr CM (2019). Hypervirulent *Klebsiella pneumoniae*. Clin Microbiol Rev.

[15] Choi M, Tennant SM, Simon R, Cross AS (2019). Progress towards the development of Klebsiella vaccines. Expert Rev Vaccines.

[16] Follador R, Heinz E, Wyres KL, Ellington MJ, Kowarik M (2016). The diversity of *Klebsiella pneumoniae* surface polysaccharides. Microb Genom.

[17] Wyres KL, Wick RR, Gorrie C, Jenney A, Follador R (2016). Identification of *Klebsiella* capsule synthesis loci from whole genome data. Microb Genom.

[18] Choi M, Hegerle N, Nkeze J, Sen S, Jamindar S (2020). The diversity of lipopolysaccharide (O) and capsular polysaccharide (K) antigens of invasive *Klebsiella pneumoniae* in a multi-country collection. Front Microbiol.

[19] Long SW, Olsen RJ, Eagar TN, Beres SB, Zhao P (2017). Population genomic analysis of 1,777 extended-spectrum beta-lactamase-producing *Klebsiella pneumoniae* isolates, Houston, Texas: Unexpected Abundance of Clonal Group 307. mBio.

[20] David S, Reuter S, Harris SR, Glasner C, Feltwell T (2019). Epidemic of carbapenem-resistant *Klebsiella pneumoniae* in Europe is driven by nosocomial spread. Nat Microbiol.

[21] Lewis J, Mphasa M, Banda R, Beale MA, Heinz E Dynamics of gut mucosal colonisation with extended spectrum beta-lactamase producing Enterobacterales in malawi. medRxiv.

[22] Malawi National Statistical Office (2019). 2018 Malawi Population and Housing Census Main Report. Zomba.

[23] World Health Organisation (2016). Consolidated Guidelines On the Use of Antiretroviral Drugs for Treating and Preventing HIV Infection: Recommendations for a Public Health Approach. Second Edition.

[24] Wood DE, Salzberg SL (2014). Kraken: ultrafast metagenomic sequence classification using exact alignments. Genome Biol.

[25] Bankevich A, Nurk S, Antipov D, Gurevich AA, Dvorkin M (2012). SPAdes: a new genome assembly algorithm and its applications to single-cell sequencing. J Comput Biol.

[26] Page AJ, De Silva N, Hunt M, Quail MA, Parkhill J (2016). Robust high-throughput prokaryote *de novo* assembly and improvement pipeline for Illumina data. Microb Genom.

[27] Gurevich A, Saveliev V, Vyahhi N, Tesler G (2013). QUAST: quality assessment tool for genome assemblies. Bioinformatics.

[28] Parks DH, Imelfort M, Skennerton CT, Hugenholtz P, Tyson GW (2015). CheckM: assessing the quality of microbial genomes recovered from isolates, single cells, and metagenomes. Genome Res.

[29] Seemann T (2014). Prokka: rapid prokaryotic genome annotation. Bioinformatics.

[30] Page AJ, Cummins CA, Hunt M, Wong VK, Reuter S (2015). Roary: rapid large-scale prokaryote pan genome analysis. Bioinformatics.

[31] Page AJ, Taylor B, Delaney AJ, Soares J, Seemann T (2016). SNP*-*sites: rapid efficient extraction of SNPs from multi-FASTA alignments. Microb Genom.

[32] Nguyen L-T, Schmidt HA, von Haeseler A, Minh BQ (2015). IQ-TREE: a fast and effective stochastic algorithm for estimating maximum-likelihood phylogenies. Mol Biol Evol.

[33] Yu G, Smith DK, Zhu H, Guan Y, Lam T-Y (2017). GGTREE: an R package for visualization and annotation of phylogenetic trees with their covariates and other associated data. Methods Ecol Evol.

[34] Lam MMC, Wick RR, Watts SC, Cerdeira LT, Wyres KL (2021). A genomic surveillance framework and genotyping tool for *Klebsiella pneumoniae* and its related species complex. Nat Commun.

[35] Hunt M, Mather AE, Sánchez-Busó L, Page AJ, Parkhill J (2017). ARIBA: rapid antimicrobial resistance genotyping directly from sequencing reads. Microb Genom.

[36] Liu B, Zheng D, Jin Q, Chen L, Yang J (2019). VFDB 2019: a comparative pathogenomic platform with an interactive web interface. Nucleic Acids Res.

[37] Diancourt L, Passet V, Verhoef J, Grimont PAD, Brisse S (2005). Multilocus sequence typing of *Klebsiella pneumoniae* nosocomial isolates. J Clin Microbiol.

[38] Inouye M, Dashnow H, Raven L-A, Schultz MB, Pope BJ (2014). SRST2: rapid genomic surveillance for public health and hospital microbiology labs. Genome Med.

[39] Alcock BP, Raphenya AR, Lau TTY, Tsang KK, Bouchard M (2020). CARD 2020: antibiotic resistome surveillance with the comprehensive antibiotic resistance database. Nucleic Acids Res.

[40] Cornick J, Musicha P, Peno C, Saeger E, Toh PI (2020). Genomic investigation of a suspected multi-drug resistant *Klebsiella pneumoniae* outbreak in a neonatal care unit in sub-Saharan Africa. bioRxiv.

[41] Musicha P, Msefula CL, Mather AE, Chaguza C, Cain AK (2019). Genomic analysis of *Klebsiella pneumoniae* isolates from Malawi reveals acquisition of multiple ESBL determinants across diverse lineages. J Antimicrob Chemother.

[42] Henson SP, Boinett CJ, Ellington MJ, Kagia N, Mwarumba S (2017). Molecular epidemiology of *Klebsiella pneumoniae* invasive infections over a decade at Kilifi County Hospital in Kenya. Int J Med Microbiol.

[43] Lewis J (2021). https://joelewis101.github.io/blantyreESBL/.

[44] Ellington MJ, Heinz E, Wailan AM, Dorman MJ, de Goffau M (2019). Contrasting patterns of longitudinal population dynamics and antimicrobial resistance mechanisms in two priority bacterial pathogens over 7 years in a single center. Genome Biol.

[45] Heinz E, Ejaz H, Bartholdson Scott J, Wang N, Gujaran S (2019). Resistance mechanisms and population structure of highly drug resistant *Klebsiella* in Pakistan during the introduction of the carbapenemase NDM-1. Sci Rep.

[46] Heinz E, Brindle R, Morgan-McCalla A, Peters K, Thomson NR (2019). Caribbean multi-centre study of *Klebsiella pneumoniae*: whole-genome sequencing, antimicrobial resistance and virulence factors. Microb Genom.

[47] (2021). Kaptive Web: User-Friendly Capsule and Lipopolysaccharide Serotype Prediction for Klebsiella Genomes | Journal of Clinical Microbiology. https://jcm.asm.org/content/56/6/e00197-18.

[48] Strahilevitz J, Jacoby GA, Hooper DC, Robicsek A (2009). Plasmid-mediated quinolone resistance: a multifaceted threat. Clin Microbiol Rev.

[49] Andrade LN, Novais Â, Stegani LMM, Ferreira JC, Rodrigues C (2018). Virulence genes, capsular and plasmid types of multidrug-resistant CTX-M(-2, -8, -15) and KPC-2-producing *Klebsiella pneumoniae* isolates from four major hospitals in Brazil. Diagn Microbiol Infect Dis.

[50] Lewis JM, Lester R, Mphasa M, Banda R, Edwards T (2020). Emergence of carbapenemase-producing *Enterobacteriaceae* in Malawi. J Glob Antimicrob Resist.

[51] Kumwenda GP, Sugawara Y, Abe R, Akeda Y, Kasambara W (2019). First identification and genomic characterization of multidrug-resistant carbapenemase-producing *Enterobacteriaceae* clinical isolates in Malawi, Africa. J Med Microbiol.

[52] Lipworth S, Vihta K-D, Chau KK, Kavanagh J, Davies T (2021). Ten years of population-level genomic *Escherichia coli* and *Klebsiella pneumoniae* Serotype surveillance informs vaccine development for invasive infections. Clin Infect Dis.

