# Supplementary material for: Genomic and antigenic diversity of colonizing Klebsiella pneumoniae isolates mirrors that of invasive isolates in Blantyre, Malawi
Source: Microb Genom. 2022 Mar 18;8(3):000778. doi: 10.1099/mgen.0.000778 (PMC9176273; doi:10.1099/mgen.0.000778)
Supplement: Supplementary material 1 [file mgen-8-0778-s001.pdf]

Genomic and antigenic diversity of colonising *Klebsiella pneumoniae* isolates mirrors that of invasive isolates in Blantyre, Malawi

Supplementary Material

Supplementary Table 1: Distribution of O-antigens

O-type	n (%)	Cumulative n (%)
O1v1	60 (29.6%)	60 (29.6%)
O2v2	30 (14.8%)	90 (44.3%)
O1v2	29 (14.3%)	119 (58.6%)
O4	29 (14.3%)	148 (72.9%)
O3b	16 (7.9%)	164 (80.8%)
O2v1	13 (6.4%)	177 (87.2%)
O5	6 (3.0%)	183 (90.1%)
OL101	6 (3.0%)	189 (93.1%)
OL103	6 (3.0%)	195 (96.1%)
Unknown	6 (3.0%)	201 (99.0%)
O12	1 (0.5%)	202 (99.5%)
OL104	1 (0.5%)	203 (100.0%)

Supplementary Table 2: Distribution of K-antigens

K-type	n (%)	Cumulative n (%)
KL2	23 (11.3%)	23 (11.3%)
KL102	19 (9.4%)	42 (20.7%)
KL15	13 (6.4%)	55 (27.1%)
KL25	12 (5.9%)	67 (33.0%)
KL111	11 (5.4%)	78 (38.4%)
Unknown	9 (4.4%)	87 (42.9%)
KL149	8 (3.9%)	95 (46.8%)
KL30	8 (3.9%)	103 (50.7%)
KL16	7 (3.4%)	110 (54.2%)
KL24	7 (3.4%)	117 (57.6%)
KL10	6 (3.0%)	123 (60.6%)
KL17	6 (3.0%)	129 (63.5%)
KL57	6 (3.0%)	135 (66.5%)
KL112	5 (2.5%)	140 (69.0%)
KL23	5 (2.5%)	145 (71.4%)
KL27	5 (2.5%)	150 (73.9%)
KL110	4 (2.0%)	154 (75.9%)
KL48	4 (2.0%)	158 (77.8%)
KL137	3 (1.5%)	161 (79.3%)
KL18	3 (1.5%)	164 (80.8%)
KL39	3 (1.5%)	167 (82.3%)
KL108	2 (1.0%)	169 (83.3%)
KL13	2 (1.0%)	171 (84.2%)
KL136	2 (1.0%)	173 (85.2%)
KL151	2 (1.0%)	175 (86.2%)
KL155	2 (1.0%)	177 (87.2%)
KL21	2 (1.0%)	179 (88.2%)
KL54	2 (1.0%)	181 (89.2%)
KL63	2 (1.0%)	183 (90.1%)
KL67	2 (1.0%)	185 (91.1%)
KL8	2 (1.0%)	187 (92.1%)
KL103	1 (0.5%)	188 (92.6%)
KL105	1 (0.5%)	189 (93.1%)
KL114	1 (0.5%)	190 (93.6%)
KL115	1 (0.5%)	191 (94.1%)
KL12	1 (0.5%)	192 (94.6%)
KL123	1 (0.5%)	193 (95.1%)
KL14	1 (0.5%)	194 (95.6%)
KL145	1 (0.5%)	195 (96.1%)
KL157	1 (0.5%)	196 (96.6%)
KL19	1 (0.5%)	197 (97.0%)
KL52	1 (0.5%)	198 (97.5%)
KL59	1 (0.5%)	199 (98.0%)
KL62	1 (0.5%)	200 (98.5%)
KL64	1 (0.5%)	201 (99.0%)
KL74	1 (0.5%)	202 (99.5%)
KL9	1 (0.5%)	203 (100.0%)

Supplementary Table 3: Fisher's exact test Benjamini-Hochberg corrected p-values testing equal distribution of O-types across invasive and carriage isolates

**O-type**	**Uncorrected p-value**	**Benjamini-Hochberg corrected p-values**
O1/O2v1	0.15400	0.431
O12	1.00000	1.000
O1v1	0.20300	0.474
O1v2	0.04140	0.290
O2v1	0.29800	0.596
O2v2	0.53000	0.824
O3/O3a	0.00906	0.127
O3b	0.09730	0.341
O4	0.75500	0.903
O5	0.77400	0.903
OL101	0.48800	0.824
OL103	0.08520	0.341
OL104	1.00000	1.000
Unknown	0.75800	0.903

Supplementary Table 4: Fisher's exact test Benjamini-Hochberg corrected p-values testing equal distribution of K-types across invasive and carriage isolates

K-type	Uncorrected p-value	Benjamini-Hochberg corrected p-values
KL10	0.154000	0.6640
KL102	0.054300	0.5120
KL103	1.000000	1.0000
KL104	0.394000	0.8390
KL105	1.000000	1.0000
KL106	1.000000	1.0000
KL108	0.521000	0.8820
KL109	0.394000	0.8390
KL110	0.157000	0.6640
KL111	0.032300	0.3550
KL112	0.161000	0.6640
KL114	1.000000	1.0000
KL115	1.000000	1.0000
KL12	1.000000	1.0000
KL122	0.394000	0.8390
KL123	1.000000	1.0000
KL125	0.394000	0.8390
KL127	0.394000	0.8390
KL13	1.000000	1.0000
KL132	1.000000	1.0000
KL134	0.394000	0.8390
KL136	0.521000	0.8820
KL137	0.282000	0.8390
KL14	0.564000	0.9080
KL142	0.394000	0.8390
KL145	1.000000	1.0000
KL149	0.094500	0.6640

KL15	0.013900	0.2290
KL151	0.521000	0.8820
KL155	0.521000	0.8820
KL157	1.000000	1.0000
KL158	0.394000	0.8390
KL16	0.774000	1.0000
KL165	0.394000	0.8390
KL17	1.000000	1.0000
KL18	0.282000	0.8390
KL19	1.000000	1.0000
KL2	0.865000	1.0000
KL20	0.003490	0.0768
KL21	0.521000	0.8820
KL23	0.252000	0.8390
KL24	0.538000	0.8880
KL25	0.176000	0.6830
KL27	0.085200	0.6640
KL3	0.154000	0.6640
KL30	0.770000	1.0000
KL39	0.652000	1.0000
**KL43**	**0.000194**	**0.0128**
KL45	0.154000	0.6640
KL48	0.652000	1.0000
KL5	0.394000	0.8390
KL51	0.154000	0.6640
KL52	1.000000	1.0000
KL53	0.394000	0.8390
KL54	0.521000	0.8820
KL55	0.154000	0.6640
KL57	1.000000	1.0000
KL59	1.000000	1.0000
**KL62**	**0.001280**	**0.0422**
KL63	1.000000	1.0000
KL64	1.000000	1.0000
KL67	0.521000	0.8820
KL74	1.000000	1.0000
KL8	0.521000	0.8820
KL9	1.000000	1.0000
Unknown	0.028400	0.3550

Supplementary Table 5: Distribution of Kleborate-identified virulence loci stratified by invasive versus colonising isolates in the full collection (top) and sensitivity analysis population (bottom).

	Locus present in		
Virulence locus	Invasive Isolates	Colonising Isolates	p value
**All samples**			
clb	4/139 (3%)	0/214 (0%)	0.023
iro	8/139 (6%)	1/214 (0%)	0.003
iuc	18/139 (13%)	1/214 (0%)	<0.001
rmpA	3/139 (2%)	1/214 (0%)	0.304
rmpA2	18/139 (13%)	1/214 (0%)	<0.001
ybt	26/139 (19%)	68/214 (32%)	0.007
**Sensitivity analysis population**			
clb	4/78 (5%)	0/191 (0%)	0.007
iro	7/78 (9%)	1/191 (1%)	0.001
iuc	7/78 (9%)	1/191 (1%)	0.001
rmpA	3/78 (4%)	1/191 (1%)	0.075
rmpA2	7/78 (9%)	1/191 (1%)	0.001
ybt	21/78 (27%)	61/191 (32%)	0.467

Supplementary Table 6: Distribution of VFDB-defined virulence loci stratified by invasive versus colonising isolates in the full collection (top) and sensitivity analysis population (bottom).

Virulence lcous	Identfied genes	All isolates	Invasive isolates	Colonising Isolates	p-value
**All samples**					
AcrAB	*acrA, acrB*	350/353 (99%)	136/139 (98%)	214/214 (100%)	0.060
Regulation of capsule synthesis	*rcsA, rcsB*	350/353 (99%)	136/139 (98%)	214/214 (100%)	0.060
Type 1 fimbriae	*fimA, fimB, fimC, fimE, fimH, fimI*	348/353 (99%)	137/139 (99%)	211/214 (99%)	1.000
Type 3 fimbriae	*mrkA, mrkB, mrkC, mrkD, mrkF, mrkH, mrkI, mrkJ*	347/353 (98%)	135/139 (97%)	212/214 (99%)	0.217
T6SS	*clpV/tssH, dotU/tssL, hcp/tssD, icmF/tssM, impA/tssA, KPHS_23120, sciN/tssJ, tli1, tssF, tssG, vasE/tssK, tle1, vgrG/tssI, vipA/tssB, vipB/tssC*	338/353 (96%)	134/139 (96%)	204/214 (95%)	0.789
E. coli common pilus	*yagW/ecpD, yagY/ecpB, yagZ/ecpA, ykgK/ecpR*	32/353 (9%)	2/139 (1%)	30/214 (14%)	<0.001
Allantion utilization	*allA, allB, allC, allD, allR, allS*	12/353 (3%)	4/139 (3%)	8/214 (4%)	0.770
E. coli K1 capsule	*kpsD, kpsE, kpsF*	2/353 (1%)	2/139 (1%)	0/214 (0%)	0.154
P fimbriae	*papI*	2/353 (1%)	2/139 (1%)	0/214 (0%)	0.154
Afa/Dr family	*afaE-I*	1/353 (0%)	1/139 (1%)	0/214 (0%)	0.394
Dr adhesins	*draP*	1/353 (0%)	1/139 (1%)	0/214 (0%)	0.394
E. coli heme uptake	*chuA, chuS, chuT, chuU, chuV, chuW, chuX, chuY*	1/353 (0%)	1/139 (1%)	0/214 (0%)	0.394
Flagella	*fliJ*	1/353 (0%)	1/139 (1%)	0/214 (0%)	0.394
Invasion of brain endothelial cells	*ibeA*	1/353 (0%)	1/139 (1%)	0/214 (0%)	0.394
Secreted autotransporter toxin	*sat*	1/353 (0%)	1/139 (1%)	0/214 (0%)	0.394
Shigella enterotoxin 2	*senB*	1/353 (0%)	1/139 (1%)	0/214 (0%)	0.394
Type II secretion system	*gspD, gspK*	1/353 (0%)	1/139 (1%)	0/214 (0%)	0.394
Sensitivity analysis population					
AcrAB	*acrA, acrB*	269/269 (100%)	78/78 (100%)	191/191 (100%)	1.000

Regulation of capsule synthesis	*rcsA, rcsB*	269/269 (100%)	78/78 (100%)	191/191 (100%)	1.000
Type 1 fimbriae	*fimA, fimB, fimC, fimE, fimH, fimI*	266/269 (99%)	78/78 (100%)	188/191 (98%)	0.559
Type 3 fimbriae	*mrkA, mrkB, mrkC, mrkD, mrkF, mrkH, mrkI, mrkJ*	266/269 (99%)	77/78 (99%)	189/191 (99%)	1.000
T6SS	*clpV/tssH, dotU/tssL, hcp/tssD, icmF/tssM, impA/tssA, KPHS_23120, sciN/tssJ, tli1, tssF, tssG, vasE/tssK, tle1, vgrG/tssI, vipA/tssB, vipB/tssC*	258/269 (96%)	76/78 (97%)	182/191 (95%)	0.519
E. coli common pilus	*yagW/ecpD, yagY/ecpB, yagZ/ecpA, ykgK/ecpR*	27/269 (10%)	2/78 (3%)	25/191 (13%)	0.007
Allantion utilization	*allA, allB, allC, allD, allR, allS*	11/269 (4%)	3/78 (4%)	8/191 (4%)	1.000
Afa/Dr family	*afaE-I*	1/269 (0%)	1/78 (1%)	0/191 (0%)	0.290
Dr adhesins	*draP*	1/269 (0%)	1/78 (1%)	0/191 (0%)	0.290
E. coli heme uptake	*chuA, chuS, chuT, chuU, chuV, chuW, chuX, chuY*	1/269 (0%)	1/78 (1%)	0/191 (0%)	0.290
Invasion of brain endothelial cells	*ibeA*	1/269 (0%)	1/78 (1%)	0/191 (0%)	0.290
E. coli K1 capsule	*kpsD, kpsE, kpsF*	1/269 (0%)	1/78 (1%)	0/191 (0%)	0.290
P fimbriae	*papI*	1/269 (0%)	1/78 (1%)	0/191 (0%)	0.290
Secreted autotransporter toxin	*sat*	1/269 (0%)	1/78 (1%)	0/191 (0%)	0.290
Shigella enterotoxin 2	*senB*	1/269 (0%)	1/78 (1%)	0/191 (0%)	0.290
Type II secretion system	*gspD, gspK*	1/269 (0%)	1/78 (1%)	0/191 (0%)	0.290
Flagella	*fliJ*	0/269 (0%)	0/78 (0%)	0/191 (0%)	1.000

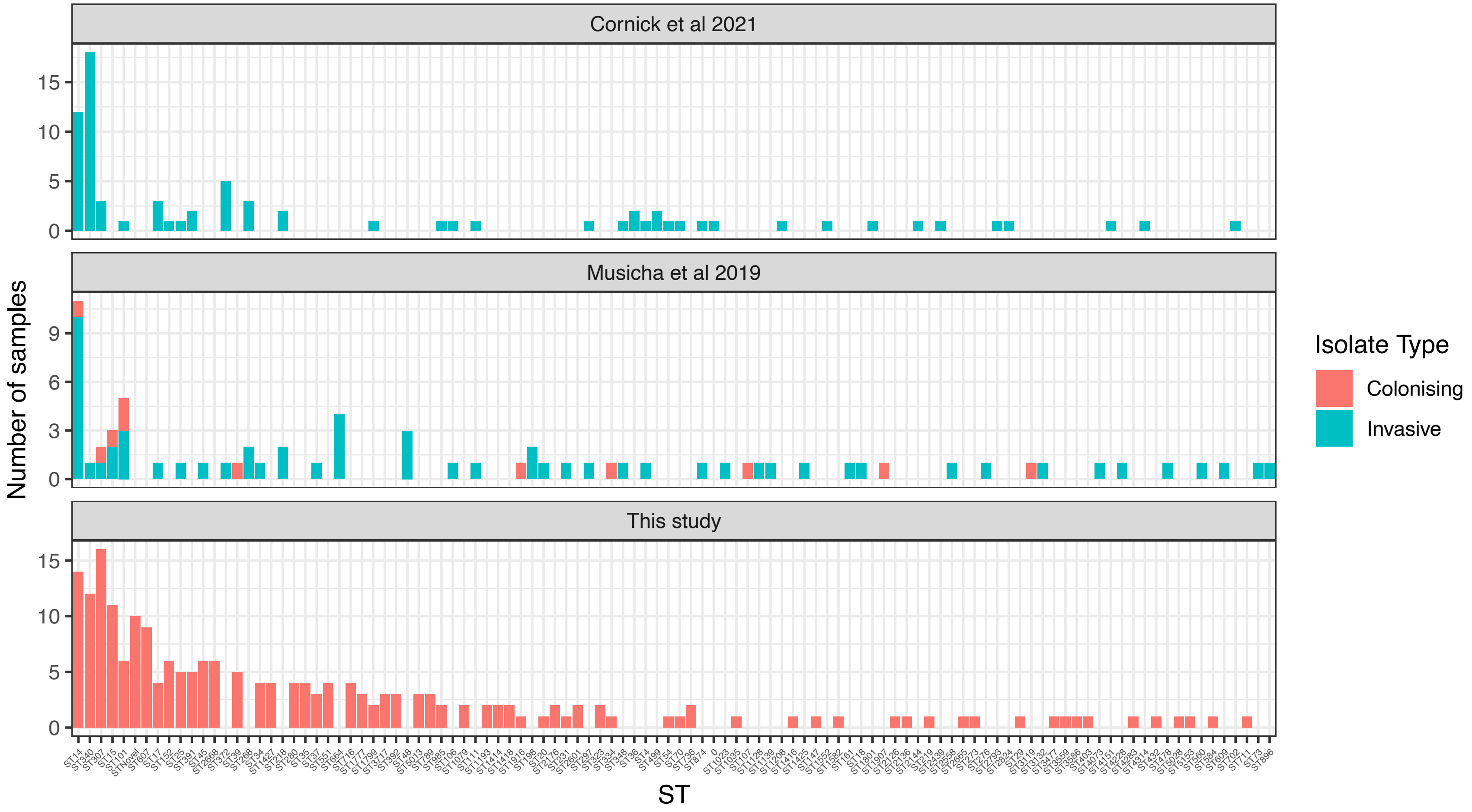

Supplementary Figure 1: Multilocus sequence type (ST) distribution of all included Malawian genomes.

A

B

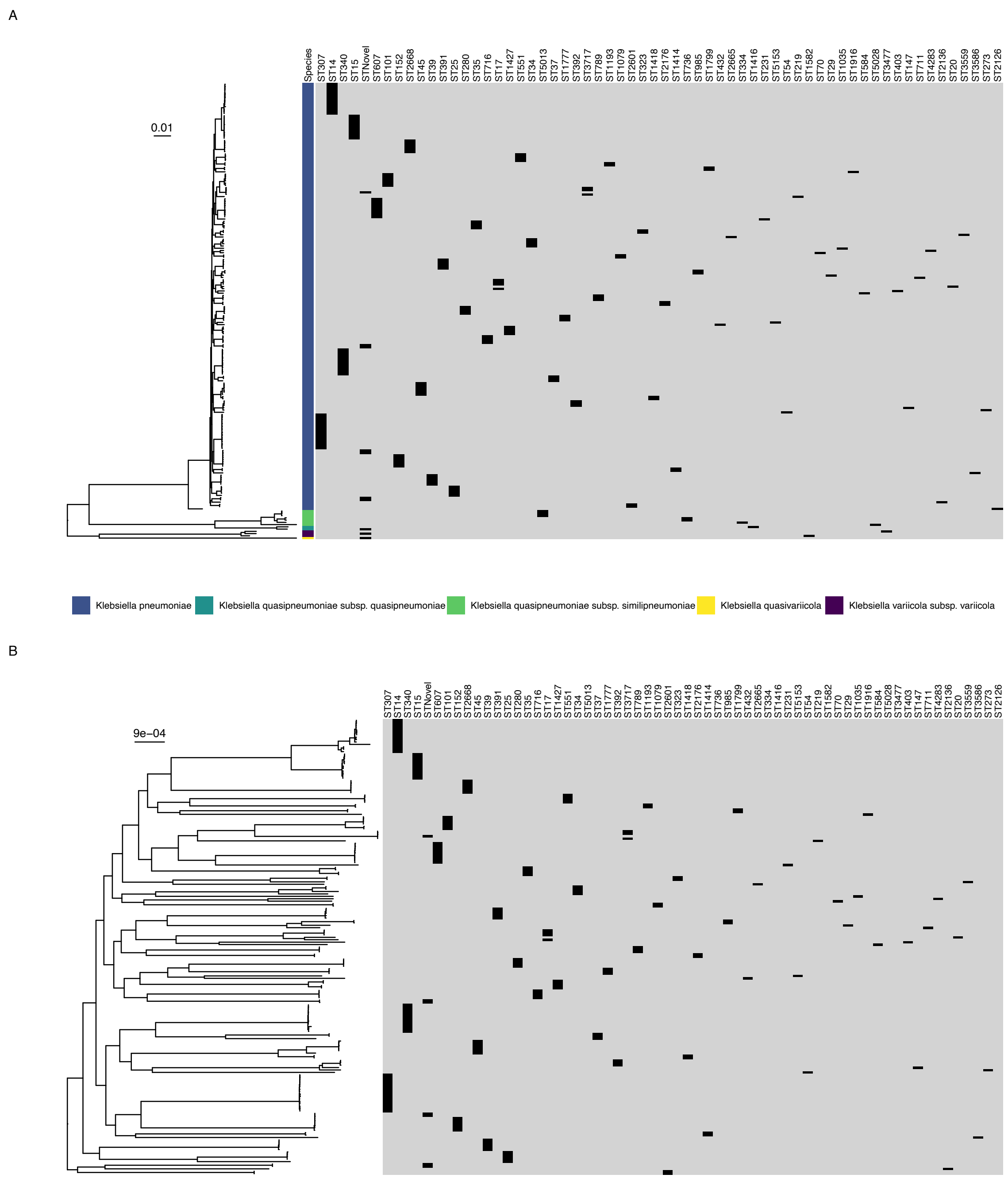

Supplementary Figure 2: Midpoint-rooted core-gene maximum-likelihood phylogenetic trees of isolates from the study. (A) shows the full phylogeny with species (coloured bar) and sequence type (ST, black bars); (B) restricts to *K. pneumoniae subsp. pneumoniae*, showing STs (black bars). Scale bars show nucleotide substitutions per site.

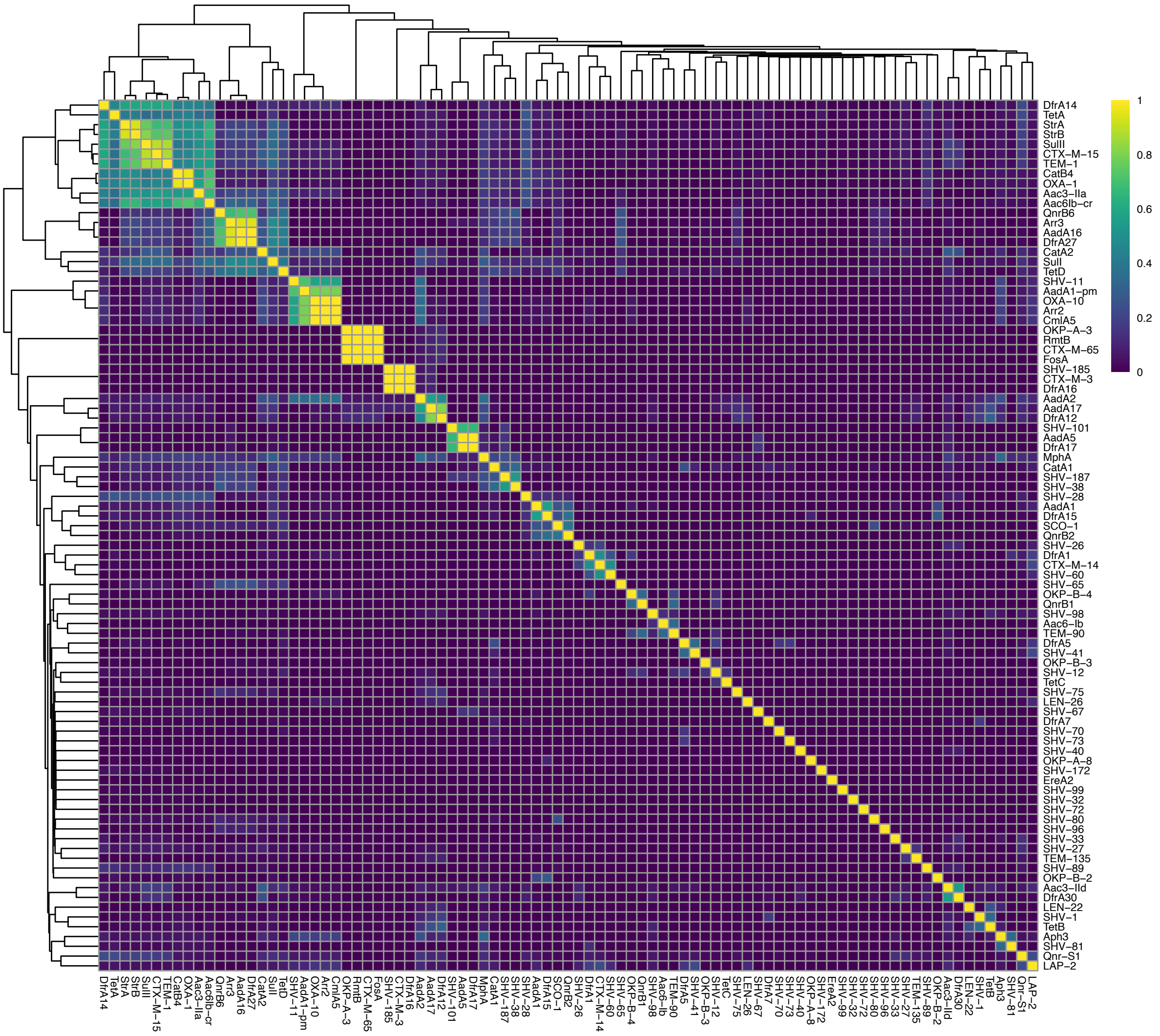

Supplementary Figure 3: Jaccard-distance heatmap of presence of ARIBA-identified AMR genes, clustered with a hierarchical clustering algorithm. Several AMR-gene clusters are apparent.

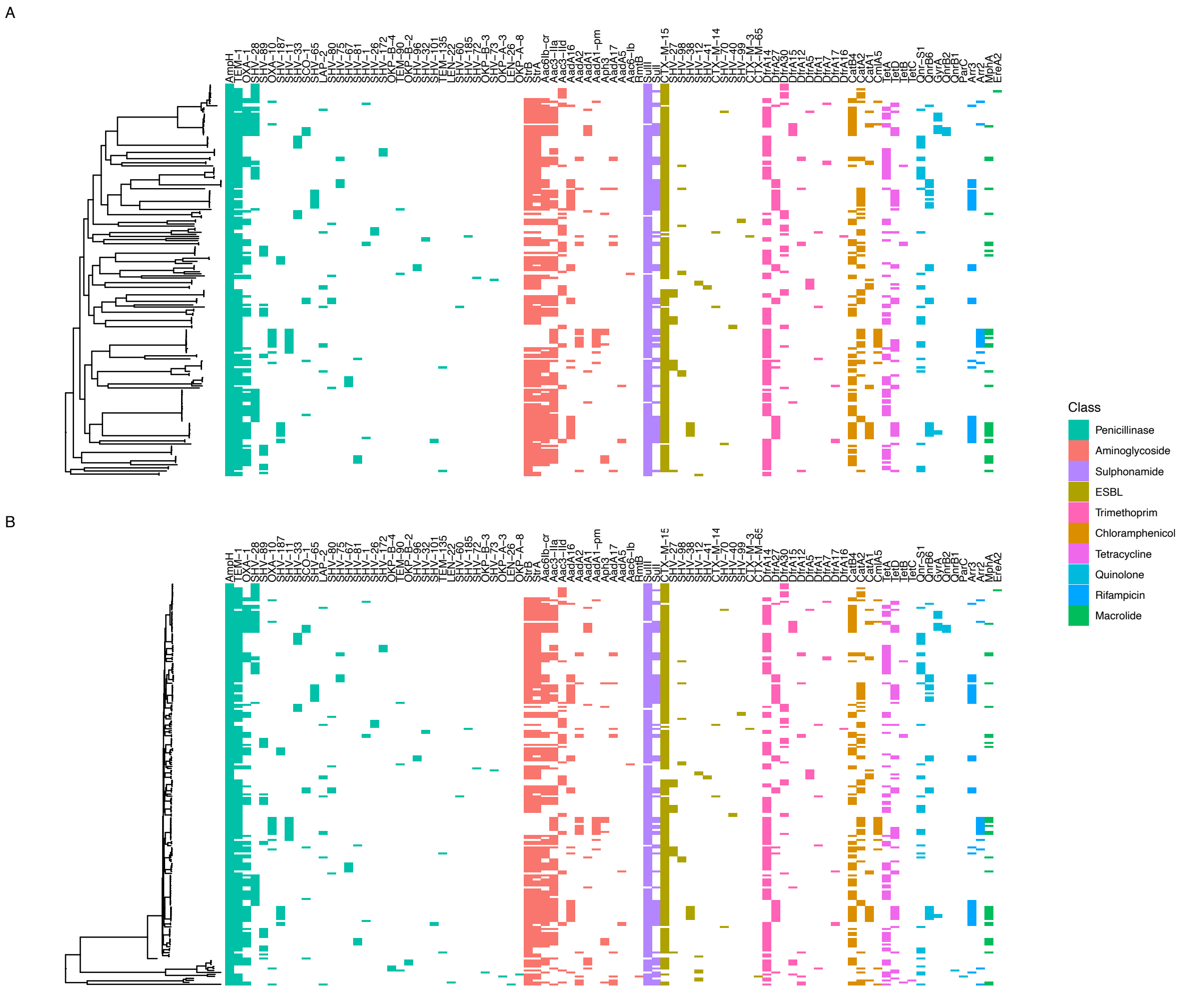

Supplementary Figure 4: Presence of ARIBA-identified AMR genes mapped back to phylogeny for (A) KPI isolates only (B) all samples. Some lineage association of AMR genes is apparent.

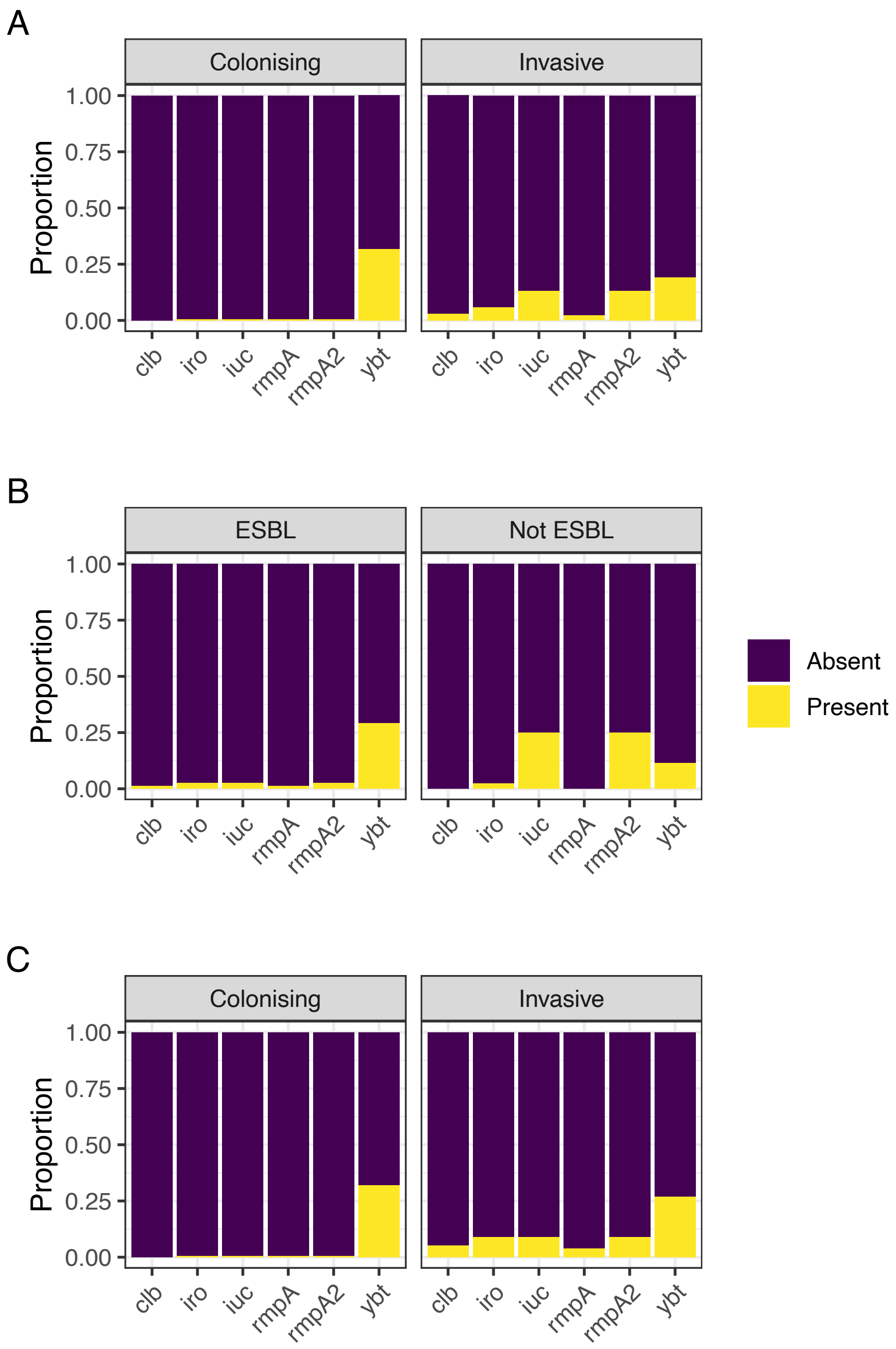

Supplementary Figure 5: Virulence determinants of Malawian isolates stratified by (A) carriage or infecting isolate (B) ESBL or non-ESBL isolate

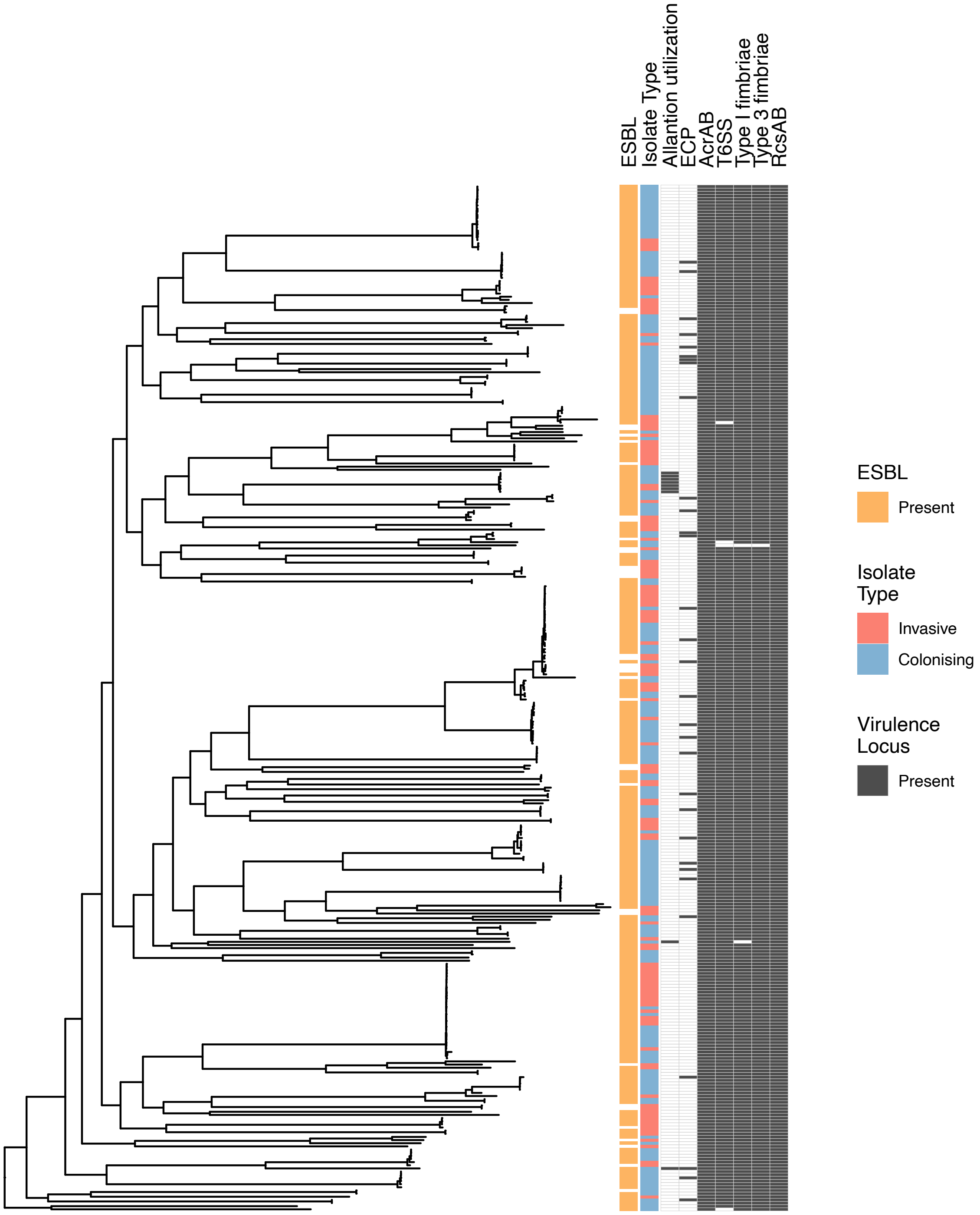

Supplementary Figure 6: Midpoint rooted core-gene maximum-likelihood phylogenetic tree of Malawian isolates, showing VFDB-identified virulence determinants. Included are all genomes from this study and context genomes from Malawian studies (n = 353), and restricted to *Klebsiella pneumoniae subsp. pneumoniae.*

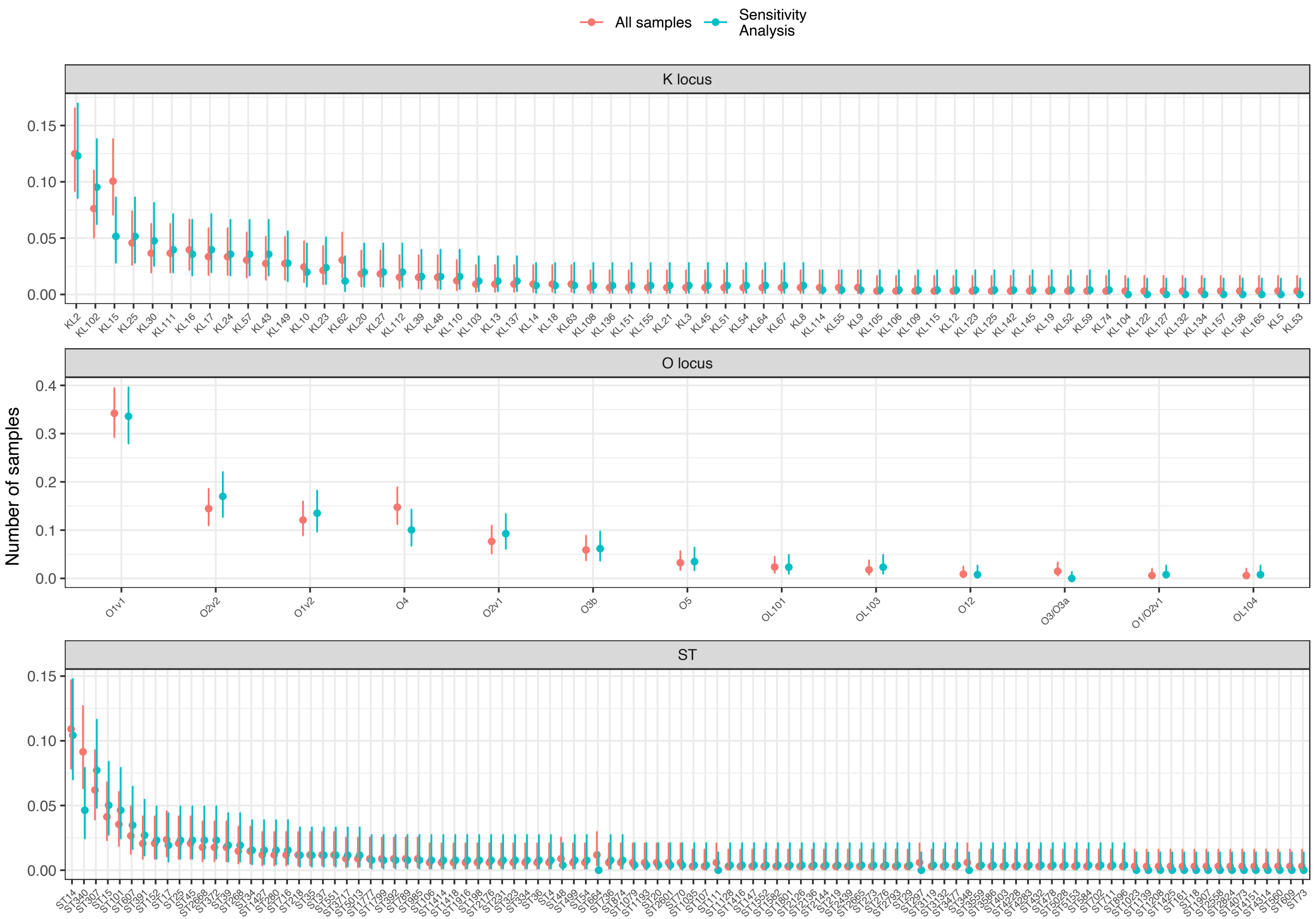

Supplementary Figure 7: K-, O-locus and ST distribution in all samples and in sensitivity analysis population

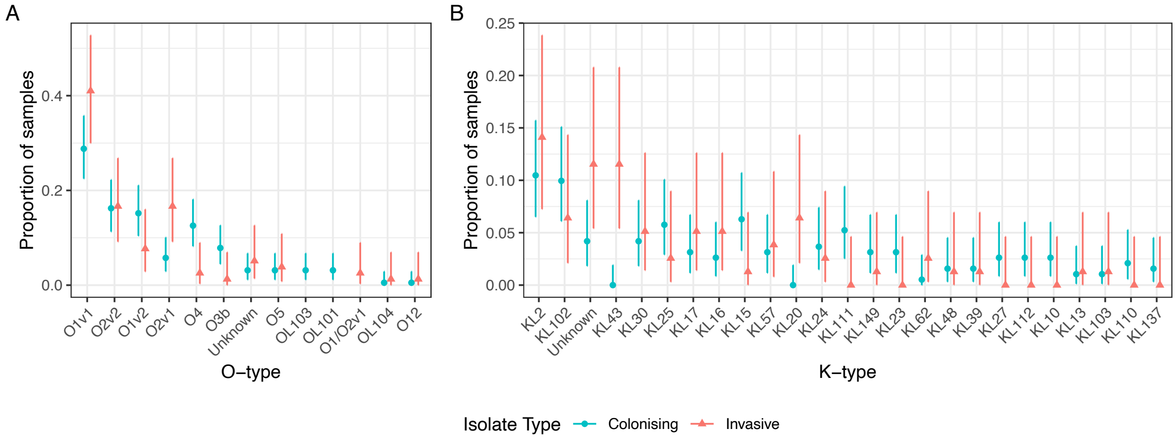

Supplementary Figure 8: O- and K- type distribution in colonising and infecting isolates in the sensitivity analysis population
